# Xeno- and feeder-free differentiation of human pluripotent stem cells to two distinct ocular epithelial cell types using simple modifications of one method

**DOI:** 10.1186/s13287-017-0738-4

**Published:** 2017-12-29

**Authors:** Heidi Hongisto, Tanja Ilmarinen, Meri Vattulainen, Alexandra Mikhailova, Heli Skottman

**Affiliations:** 1 BioMediTech Institute, Faculty of Medicine and Life Sciences, University of Tampere, Arvo Ylpön katu 34, 33520 Tampere, Finland; 2 Department of Ophthalmology, SILK, Faculty of Medicine and Life Sciences, University of Tampere, Tampere, Finland; 3 Finnish Federation of the Visually Impaired, Helsinki, Finland

**Keywords:** Human embryonic stem cells, Human induced pluripotent stem cells, Retinal pigment epithelial cells, Corneal limbal epithelial stem cells, Xeno-free, Feeder-free

## Abstract

**Background:**

Human pluripotent stem cells (hPSCs) provide a promising cell source for ocular cell replacement therapy, but often lack standardized and xenogeneic-free culture and differentiation protocols. We aimed to develop a xeno- and feeder cell-free culture system for undifferentiated hPSCs along with efficient methods to derive ocular therapy target cells: retinal pigment epithelial (RPE) cells and corneal limbal epithelial stem cells (LESCs).

**Methods:**

Multiple genetically distinct hPSC lines were adapted to a defined, xeno-, and feeder-free culture system of Essential 8™ medium and laminin-521 matrix. Thereafter, two-stage differentiation methods toward ocular epithelial cells were established utilizing xeno-free media and a combination of extracellular matrix proteins. Both differentiation methods shared the same basal elements, using only minor inductive modifications during early differentiation towards desired cell lineages. The resulting RPE cells and LESCs were characterized after several independent differentiation experiments and recovery after xeno-free cryopreservation.

**Results:**

The defined, xeno-, and feeder-free culture system provided a robust means to generate high-quality hPSCs with chromosomal stability limited to early passages. Inductive cues introduced during the first week of differentiation had a substantial effect on lineage specification, cell survival, and even mature RPE properties. Derivative RPE formed functional epithelial monolayers with mature tight junctions and expression of RPE genes and proteins, as well as phagocytosis and key growth factor secretion capacity after 9 weeks of maturation on inserts. Efficient LESC differentiation led to cell populations expressing LESC markers such as p40/p63α by day 24. Finally, we established xeno-free cryobanking protocols for pluripotent hPSCs, hPSC-RPE cells, and hPSC-LESCs, and demonstrated successful recovery after thawing.

**Conclusions:**

We propose methods for efficient and scalable, directed differentiation of high-quality RPE cells and LESCs. The two clinically relevant cell types are generated with simple inductive modification of the same basal method, followed by adherent culture, passaging, and cryobanking.

**Electronic supplementary material:**

The online version of this article (doi:10.1186/s13287-017-0738-4) contains supplementary material, which is available to authorized users.

## Background

The eye contains highly specialized cell types essential for vision. Two cell types are particularly important for maintaining ocular functions: corneal limbal epithelial stem cells (LESCs) and retinal pigment epithelial (RPE) cells. LESCs are the stem cell population located in limbal niches at the corneoscleral junction, responsible for the constant renewal of the stratified corneal epithelium [1, 2]. The RPE is a pigmented monolayer located beneath the neural retina, forming the outer blood-retinal barrier (BRB) and maintaining the photoreceptors via light absorption, phagocytosis, solute transport, and growth factor secretion [3].

Significant loss or dysfunction of either LESCs or the RPE can cause devastating, blinding diseases. RPE cell death at the macula leads to loss of photoreceptor function and results in age-related macular degeneration (AMD), the leading cause of irreversible vision loss in the elderly in industrialized countries [4]. Similarly, diseases affecting the cornea are one of the leading causes of vision loss worldwide. The most severe type of corneal blindness is limbal stem cell deficiency (LSCD), where the limbus is destroyed by acute trauma, or chronic or genetic disease [5, 6]. Insufficiency of viable LESCs results in conjunctivalization and opacification of the corneal surface, and consequently loss of vision [7]. Such severe retinal and corneal diseases are difficult to treat, but cell replacement therapy is proving to be a viable strategy [8–10].

**Fig. 1:**
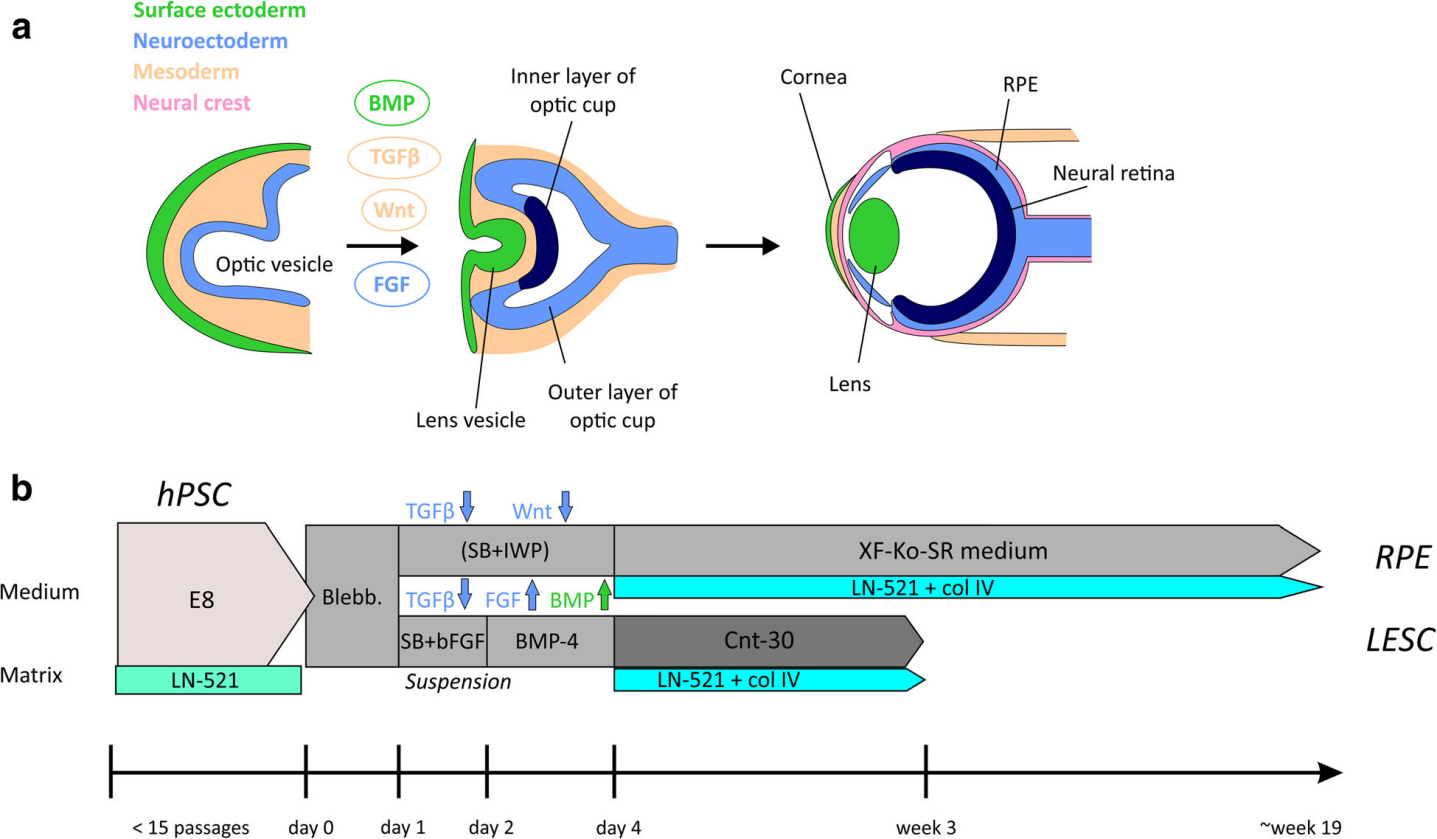
Schematic illustration of in vivo development and in vitro differentiation of RPE and LESCs. **a** At early stages of embryonic eye development, surface ectoderm thickens and invaginates together with the underlying neuroepithelium of the optic vesicle. The bilayered optic cup gives rise to the neural retina and the retinal pigment epithelium (*RPE*), while the lens and corneal epithelium develop from the surface ectoderm [14]. Some of the known signaling pathways affecting the cell fate choice during differentiation, such as bone morphogenetic protein (*BMP*) for surface ectoderm, are shown. **b** An overview of the optimized human pluripotent stem cell (*hPSC*) culture and directed RPE and limbal epithelial stem cell (*LESC*) differentiation protocols, as well as key media and matrix components used (not to scale). *Blebb.* blebbistatin, *col IV* collagen type IV, *E8* Essential 8™ Flex Medium, *FGF* fibroblast growth factor, *LN-521* recombinant laminin-521, *SB* SB-505124 hydrochloride hydrate, *TGFβ* transforming growth factor beta, *XF-Ko-SR* Knock-out™ serum replacement XenoFree CTS™

Human pluripotent stem cells (hPSCs) possess the widest differentiation potential of all stem cell types, providing endless possibilities for ocular cell replacement therapies and personalized medicine. Human embryonic stem cell (hESC) and human induced pluripotent stem cell (hiPSC)-derived RPE are already being tested in clinical trials to treat retinal degeneration [11, 12], and LSCD is a promising target for the next hPSC-based transplantation trials [13]. In the embryo, retina develops from neuroectoderm via the optic vesicle, while corneal epithelium derives from surface ectoderm [14] (Fig. 1a). In the absence of inductive cues, hPSCs spontaneously follow the neuroectodermal pathway, with a subset of cells differentiating to RPE [15]. Alternatively, sequential addition of growth factors, vitamins, and survival factors can direct hPSC differentiation towards RPE or LESC lineage [16–23]. Although many protocols have been established, they are often complex, require cell line-specific modifications, and include feeder cells or animal-derived components at some stage of hPSC culture or differentiation. Recently, strict regulatory demands and efforts toward safer cell therapy products have prompted the search for more standardized and xenogeneic-free culture and differentiation protocols. In response, some xeno-free protocols for RPE differentiation [24–28], retinal organoid culture [29], and corneal epithelial progenitor cell differentiation [30] have been introduced within the past 2 years. However, they tend to be complex and difficult to standardize across cell lines and laboratories. In this study, we aimed to set up a feeder-free, xeno-free culture method for hPSCs, and thereafter differentiation, passaging, and cryostoring protocols to obtain pure populations of high-quality RPE and LESCs. We show that a minor switch in molecular cues during early hPSC differentiation has a clear effect on the characteristics of mature RPE and on the fate choice and cell survival during LESC differentiation. Two versions of the same basal method enabled large-scale production of pure populations of high-quality RPE and LESCs that can be serially passaged and cryostored to be readily available for cell replacement therapies.

## Methods

### Pluripotent stem cell lines

The hESC lines Regea08/017 and Regea14/010 were derived in-house, and both characterized similarly to Regea08/017 as described in [31]. The hiPSC lines (UTA.04311.WT, UTA.04511.WT, UTA.04607.WT, UTA.10902.EURCCs, and UTA.10802.EURCCs) were derived and characterized at Prof. Katriina Aalto-Setälä’s laboratory at the University of Tampere as previously described for cell lines UTA.04311.WT and UTA.04511.WT [32, 33]. The hiPSC line Hel24.3 was derived and characterized at Prof. Timo Otonkoski’s laboratory at the University of Helsinki [34].

### Pluripotent stem cell culture

Standard hPSC culture was carried out on human foreskin fibroblast (hFF) feeder cells in hESC culture medium containing 20% KnockOut™ Serum Replacement (Ko-SR; Gibco, Thermo Fisher Scientific) as described previously [31]. For feeder-free culture, hPSCs were transferred onto well plates coated with 1.09 μg/cm^2^ human recombinant laminin-521 (LN-521; Biolamina, Sweden) and cultured in Essential 8™ Flex Medium (E8; Thermo Fisher Scientific) supplemented with 50 U/ml penicillin-streptomycin (Gibco, Thermo Fisher Scientific). Single cell passaging with xeno-free TrypLE™ Select Enzyme (Gibco, Thermo Fisher Scientific) was carried out twice a week onto 0.55 μg/cm^2^ LN-521, using a plating density of 40,000–50,000 cells/cm^2^. A detailed description of hPSC culture and characterization can be found in Additional file 1 (Supplementary Materials and Methods).

### Ocular epithelial differentiation

RPE and LESC differentiation protocols are schematically summarized in Fig. 1b. Undifferentiated hPSCs were detached and transferred to Corning® Costar® Ultra-Low attachment plates in XF-Ko-SR medium (KnockOut™ Dulbecoo’s modified Eagle’s medium (DMEM) supplemented with 15% KnockOut™ SR XenoFree CTS™ (XF-Ko-SR), 2 mM GlutaMAX™, 0.1 mM 2-mercaptoethanol, 1% MEM non-essential amino acids, and 50 U/ml penicillin-streptomycin (all from Gibco, Thermo Fisher Scientific)) supplemented with 5 μM or 10 μM blebbistatin (Sigma-Aldrich) or Rock inhibitor Y-27632 dihydrochloride (ROCKi; R&D Systems) to induce embryoid body (EB) formation overnight at +37 °C.

For RPE differentiation, EBs were either allowed to undergo spontaneous differentiation in 15% XF-Ko-SR medium, or were subjected to neuroectodermal induction with 10 μM SB-505124 (Sigma-Aldrich) and 10 μM IWP-2 (Merck Millipore). During the 4–6 day induction period the medium was changed daily, after which the EBs were transferred onto well plates coated with 0.75 μg/cm^2^ LN-521 and 10 μg/cm^2^ human placental collagen type IV (col IV; Sigma-Aldrich) in 15% XF-Ko-SR medium. The medium was thereafter changed three times a week. Pigmented foci were manually separated with a scalpel, dissociated with TrypLE™ Select Enzyme, and the resulting singe-cell suspension replated to culture wells coated with LN-521 and col IV. For further passaging, the RPE cells were detached with TrypLE™ Select Enzyme. For the final passage, the RPE cells were plated to similarly coated polyethylene terephthalate (PET) hanging cell culture inserts with a 1.0 μm pore size (Merck Millipore).

For LESC differentiation, EBs were subjected to surface ectodermal induction: 1 day in XF-Ko-SR medium supplemented with 10 μM SB-505124 and 50 ng/ml human basic fibroblast growth factor (bFGF; PeproTech Inc., Rocky Hill, NJ, USA), followed by 2 days in XF-Ko-SR medium supplemented with 25 ng/ml bone morphogenetic protein (BMP)-4 (PeproTech Inc.). Thereafter, the EBs were transferred onto well plates coated with 0.75 μg/cm^2^ LN-521 and 5 μg/cm^2^ col IV in a defined and serum-free medium CnT-30 (CELLnTEC Advanced Cell Systems AG, Bern, Switzerland) at a density of approximately 15 EBs per cm^2^. The cells were thereafter maintained in CnT-30, changing the medium three times a week. A detailed description of ocular epithelial differentiation methods can be found in Additional file 1 (Supplementary Materials and Methods).

### Characterization of hPSC-RPE cells

The hPSC-RPE cells were characterized after 9 weeks of maturation on PET cell culture inserts. RPE-specific gene expression was assessed with reverse transcription polymerase chain reaction (RT-PCR), and key RPE protein expression and localization was verified with immunofluorescence labeling as previously described [35]. Images were captured with an LSM 700 Confocal microscope (Carl Zeiss, Jena, Germany) using a 40× objective or 63× oil immersion objective. Retinal pigment epithelium-specific 65 kDa protein (RPE65) and Bestrophin (BEST) protein expression levels were studied with Western blotting as previously described [36]. Pigmentation intensity and RPE cell size were quantified with ImageJ Image Processing and Analysis software (https://imagej.nih.gov/ij/) tools. Transepithelial electrical resistance (TEER) was measured with a Millicell electrical resistance system volt-ohm meter (Merck Millipore). Enzyme-linked immunosorbent assay (ELISA) for pigment epithelium-derived factor (PEDF) secretion was carried out with the Human PEDF ELISA kit (BioVendor, Brno, Czech Republic) from apical media collected after 19 h incubation and analyzed at 300× dilution according to the manufacturer’s instructions. Phagocytosis assay was conducted with porcine photoreceptor outer segments (POS) by 4 h apical feeding at +37 °C (or at +4 °C for negative controls) in the presence of 10% fetal bovine serum (FBS; Gibco, Thermo Fisher Scientific), followed by labeling with anti-opsin antibody and phalloidin-tetramethylrhodamine B isothiocyanate. Z-stack images were acquired with a confocal microscope to visualize internalized POS. Details of characterization assays and antibodies can be found in Additional file 1 (Supplementary Materials and Methods).

### Characterization of hPSC-LESCs

Gene expression during early corneal differentiation was assessed with quantitative PCR (qPCR) analysis as previously described [21]. The hPSC-LESCs were characterized after 22–25 days of differentiation, and after recovery from cryopreservation (total differentiation time 26/28 and 34 days). Immunofluorescence labeling of hPSC-LESCs was performed as previously described [21]. The protein expression of p40, p63α, and paired box protein Pax-6 (PAX6) was quantified either from double-stained cytospin samples or from cell samples double-stained directly in the culture vessels. For cell counting, 10 randomly selected images were captured with 10× magnification from two separate samples. For each image, ImageJ software was used to count nuclei positive for p40, p63α, and/or PAX6, and positive expression relative to total cell number (4',6-diamidino-2-phenylindole (DAPI) stained nuclei) was calculated. Maturation of hPSC-LESCs to a stratified corneal epithelium was induced in vitro with calcium chloride supplementation as well as airlifting and indirect co-culture with 3 T3 mouse embryonic fibroblasts. The cells were visualized with confocal imaging after immunofluorescence labeling. Details of characterization assays and antibodies can be found in Additional file 1 (Supplementary Materials and Methods).

### Cryopreservation of hPSCs, hPSC-RPE cells, and hPSC-LESCs

Undifferentiated hPSCs were cryostored with pre-chilled PSC Cryopreservation Medium according to the manufacturer’s instructions, and RevitaCell™ Supplement (both from Thermo Fisher Scientific) was used for 24 h to facilitate post-thaw recovery. Early passage cells (up to feeder-free passage level 5) were detached, counted and cryostored as a single cell suspension in 1 ml aliquots of ready-to-use, xeno-free PSC Cryopreservation Medium. Cells in CryoPure tubes (Sarstedt) were placed at –80 °C in a CoolCell™ LX Freezing Container (Sigma-Aldrich) overnight, and thereafter transferred to liquid nitrogen for long-term storage. Cell were thawed quickly at room temperature, suspended to pre-warmed media, centrifuged, and resuspended again to fresh, pre-warmed media for plating.

Human PSC-RPE cells were cryopreserved in 40% XF-Ko-SR and 10% dimethyl sulfoxide (DMSO; Sigma-Aldrich) in XF-Ko-SR medium. Cells were cryopreserved in exponential growth phase 7–14 days after passaging at either RPE passage level one or two. Human PSC-LESCs were cryopreserved in 40% XF-Ko-SR and 10% DMSO in CnT-30 medium. Cells were cryopreserved and thawed as single cells suspension to their respective media, as described above for hPSCs. For hPSC-RPE, higher concentrations (1.8 μg/cm^2^ LN-521 and 10 μg/cm^2^ col IV) of extracellular matrix (ECM) coatings on PET inserts were used for recovery after cryostorage.

## Results

We aimed to establish a xeno-free and feeder-free culture method for hPSC maintenance and efficient differentiation to clinically relevant RPE cells and LESCs. This was achieved by mimicking early ocular development with small molecule induction towards desired cell lineages, followed by culture in XF-Ko-SR medium or CnT-30 medium. Importantly, a combination of LN-521 and col IV supported attachment, growth, and differentiation of both RPE and LESCs. An overview of the in vitro differentiation protocols is shown in Fig. 1b.

### LN-521 matrix in combination with E8™ medium provides a robust, xeno- and feeder-free hPSC culture system for short-term maintenance

A xeno-free, feeder-free hPSC culture system was set up using human recombinant LN-521 as an attachment matrix and E8™ as the medium. Eight individually derived, genetically independent hPSC lines (two hESC and six hiPSC lines) were transferred from culture on hFF feeder cells to feeder-free conditions (Table 1), adopting single cell passaging after the first 1 to 2 passages. Variation in capacity to adapt to the new culture system was evident between cell lines. Maximum passages in undifferentiated culture achieved for each cell line are shown in Table 1, along with the passages used for RPE and LESC differentiation.

**Table 1:** Cell lines and maximum passages for feeder-free hPSC culture and subsequent RPE and LESC differentiations

**Cell line (hESC/hiPSC)**	**Maximum FF passage**	**Cell line (hESC/hiPSC)**	**FF passages for RPE differentiation**
Regea08/017 (**hESC1**)	28 (31 (p25: 46;XX 20q tel*))	Regea08/017 (**hESC1**)	5, 8, 10, 22, 24
Regea14/010 (**hESC2**)	11	UTA.10902.EURCCs (**hiPSC1**)	5, 7
UTA.10902.EURCCs (**hiPSC1**)	10		
UTA.04607.WT (**hiPSC2**)	15		
UTA.04511.WT (**hiPSC3**)	29 (p23: 46;XY 20q11**)	**Cell line (hESC/hiPSC)**	**FF passages for LESC differentiation**
Hel24.3 (**hiPSC4**)	19	Regea08/017 (**hESC1**)	6, 6, 8
UTA.10802.EURCCs (**hiPSC5**)	4	UTA.04607.WT (**hiPSC2**)	3, 6, 13
UTA.04311.WT (**hiPSC6**)	11		

*Changes at the q telomere of chromosome 20

**Translocation at 20q11

*FF* feeder-free, *hESC* human embryonic stem cell, *hiPSC* human induced pluripotent stem cell, *LESC* limbal epithelial stem cell, *p* passage, *RPE* retinal pigment epithelium

**Fig. 2:**
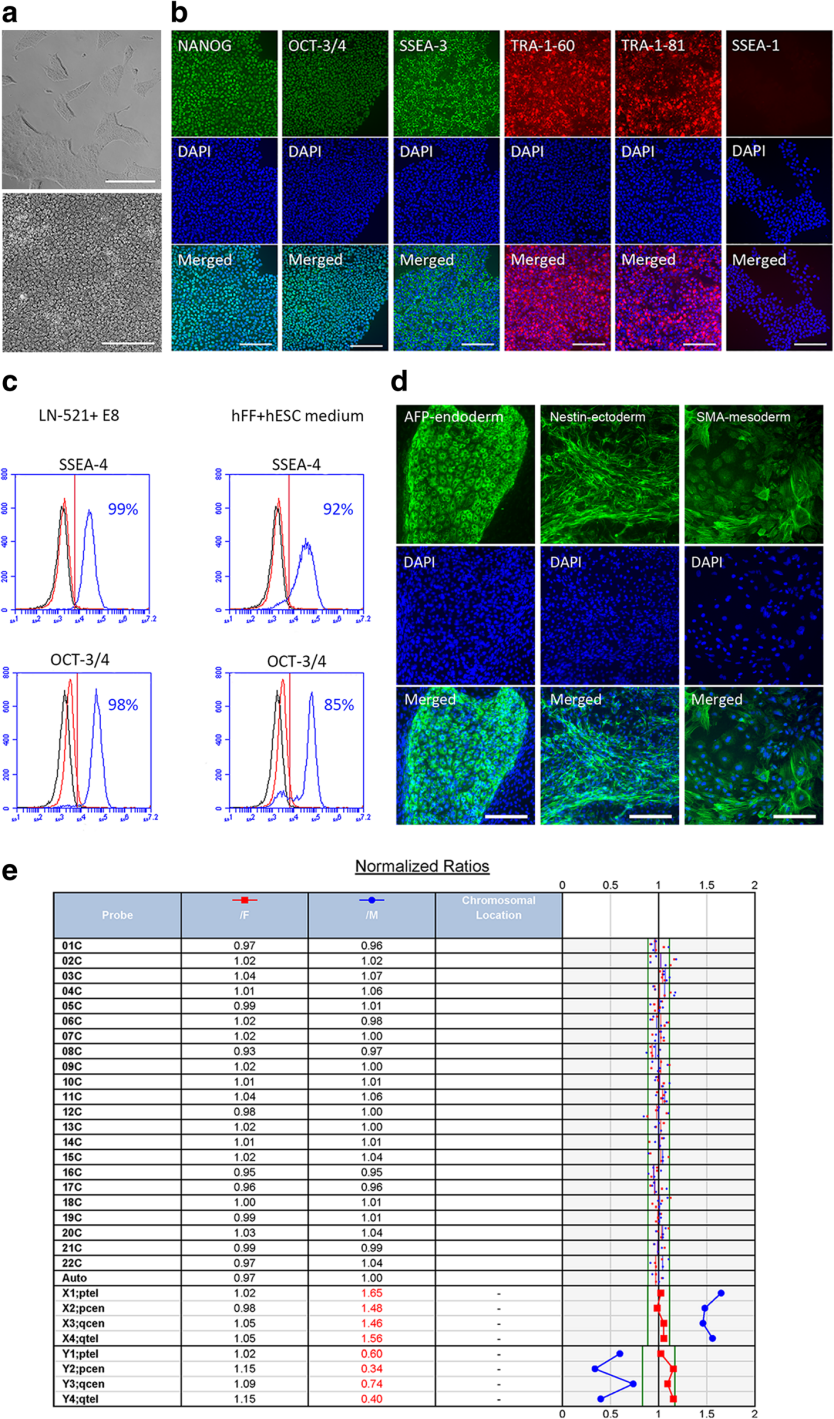
Essential hPSC characteristics were maintained throughout culture on recombinant laminin-521 (*LN-521*) in Essential 8™ Flex Medium (*E8*), shown for hESC1. **a** Typical undifferentiated colony morphology the day after passaging (*upper picture*, *scale bar* = 50 μm) and a higher magnification of the cells at confluency prior to the next passaging (day 4, *lower picture*, *scale bar* = 20 μm). Cells at feeder-free passage level 4, passage 40 in total. **b** Expression of pluripotency markers NANOG, OCT-3/4, SSEA-3, SSEA-4, TRA-1-60, and TRA-1-81, and no expression of early differentiation marker SSEA-1, at passages 8–13. Nuclei counterstained with DAPI; *scale bars* = 200 μm. **c** Flow cytometry analysis of human embryonic stem cells (*hESCs*) cultured in feeder-free conditions compared to hESCs cultured on hFF feeder cells in standard hESC medium. *Blue* histogram for positive, *black* for negative (unstained) sample, and *red* for isotype control. **d** Expression of markers of the three embryonic germ layers after spontaneous differentiation. Nuclei counterstained with DAPI; *scale bars* = 200 μm. **e** Cells after 25 passages showing normal female karyotype in KaryoLite BoBs assay. The results are shown as signal relative to karyotypically normal female (/F, *red*) and male (/M, *blue*) genomic DNA used as a reference (equal to 1) for each of the 24 chromosomal probes (cover both p and q arms of all chromosomes). Software threshold for changes shown as a *green* line and deviating results in *red*

The hPSCs grew as homogeneous monolayers with typical morphology and expression of pluripotency markers, as shown for hESC1 in Fig. 2. Comparison with hPSCs cultured on hFF feeder cells showed higher expression of stage-specific embryonic antigen (SSEA)-4 and octamer-binding transcription factor (OCT)-3/4 pluripotency markers in the feeder-free culture system (Fig. 2c). Feeder-free hPSCs were capable of spontaneous differentiation to all three embryonic germ lineages, and retained a stable karyotype at early passages (Fig. 2d and e). Characterization data for hiPSC1 and hiPSC2 are shown in Additional file 2 (Figure S1) and Additional file 3 (Figure S2), respectively. Cryostocks in xeno-free cryomedium were prepared at early passages (up to passage level 5) for each cell line and cell recovery was successful after several weeks of cryostorage.

In addition, hPSC culture was attempted in NutriStem® hPSC XF Medium combined with LN-521 matrix, but neither the hESC2 line nor the hiPSC4 line were able to grow beyond the fourth passage. E8 was also tested in combination with truncated human recombinant vitronectin (VTN-N) matrix, weekly passaging hPSCs as cell clusters with EDTA as recommended by the manufacturer. This culture system enabled undifferentiated pluripotent culture of hESC1 for 23 passages (with normal karyotype at passage 15) and hiPSC3 for 15 passages (Additional file 4: Figure S3). However, cluster passaging on VTN-N led to varying split ratios and passaging intervals, making the protocol user-sensitive, more laborious, and difficult to standardize. In contrast to colony culture on VTN-N, single cell passaging on LN-521 matrix allowed for cell counting and consistent plating densities around 50,000 cells/cm^2^, guaranteeing standardized and efficient cell production. In addition, passaging twice a week allowed for a weekend-free feeding regimen. Our post-hoc cell counting showed that, in practice, within each passage the hESC1 populations multiplied on average 3.44-fold (SD 1.71, *n* = 24 in feeder-free passages 3–12) and hiPSC2 multiplied on average 2.89-fold (SD 0.77, *n* = 11 in feeder-free passages 3–9) (Additional file 5: Table S1).

Karyotypic changes at the 20q arm occurred in hESC1 after 25 passages and in hiPSC3 after 23 passages in the LN-521 and E8 culture system. The karyotypically abnormal cells acquired a growth and differentiation advantage to RPE and the cultures were thus aborted. However, long-term, feeder-free culture of hESC1 was also carried out with a normal karyotype up to passage 27. In order to avoid chromosomal changes, hPSCs were thereafter cultured for a maximum of 15 passages in feeder-free conditions and only low passage cells were used for differentiation.

### Two-stage xeno-free differentiation yielded functional hPSC-RPE, with early neuroectodermal induction increasing pigmentation

**Fig. 3:**
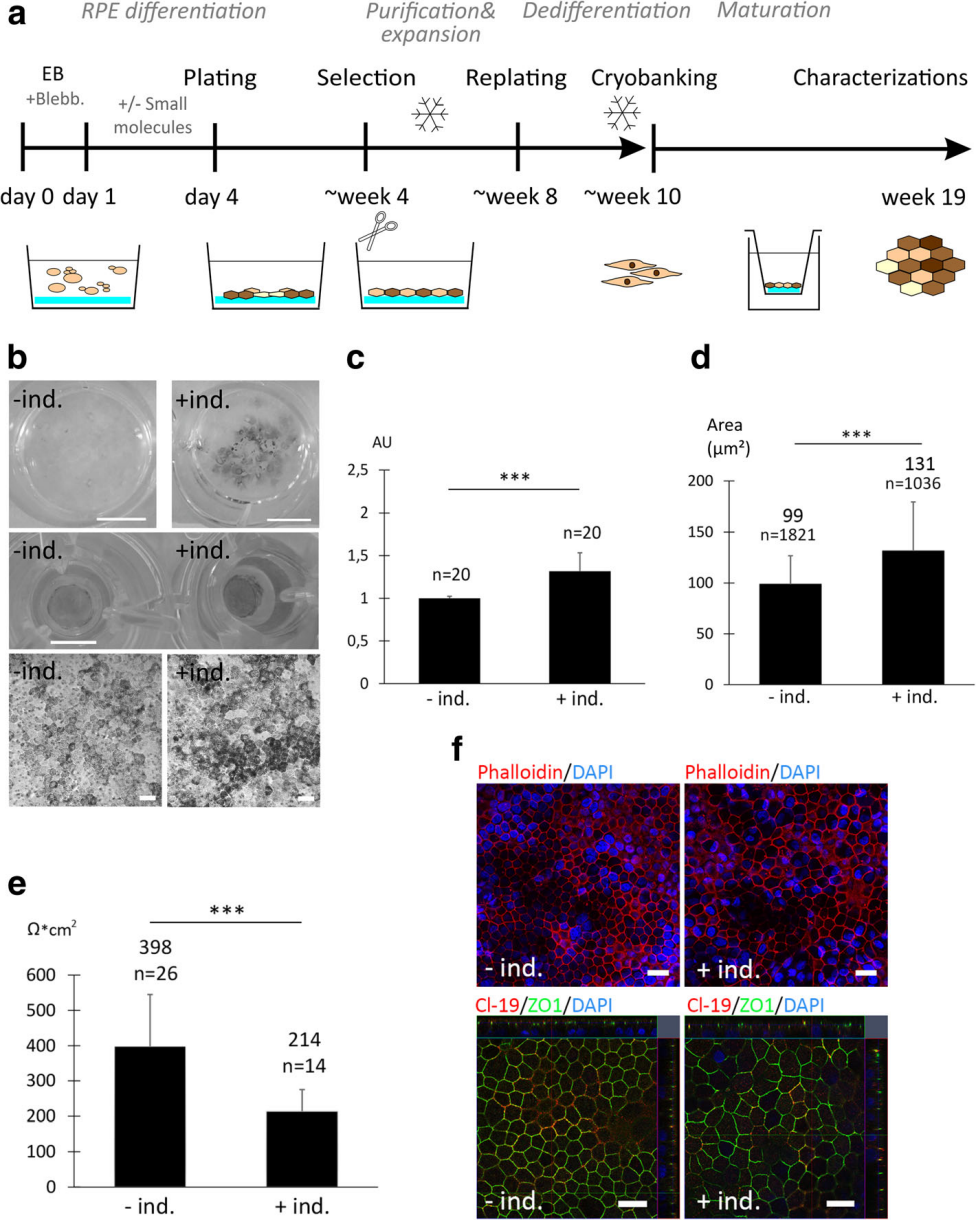
A xeno-free, feeder-free differentiation strategy led to efficient production of high-quality retinal pigment epithelial (*RPE*) cells. **a** Schematic illustration of the RPE differentiation pipeline. **b** Images of pigmented patches after 35 days of differentiation (*upper row*; *scale bars* = 1 cm) with and without initial neuroectodermal induction (*+/– ind.*) for hESC1-RPE. Images of cell culture inserts (*middle row*; *scale bar* = 6.5 mm) and DIC confocal images (*lower row*; *scale bars* = 10 μm) after 9 weeks of final maturation on inserts, illustrating the difference in pigmentation. **c** Quantification of pigmentation using image analysis after 9 weeks on inserts presented as mean pixel intensity relative to spontaneous differentiation (– ind.; *n* = images from two individual differentiation experiments), and (**d**) quantification of cell size after 9 weeks on inserts presented as mean cell area (μm^2^; *n* = number of cells examined). **e** Mean TEER values after 9 weeks on inserts, differentiated with or without induction (*n* = inserts analyzed from four individual differentiation experiments). **f** Phalloidin labeling for filamentous actin and vertical confocal sections of junctional proteins zonula occludens-1 (*ZO-1*) and Claudin-19 (*Cl-19*). *Scale bars* = 20 μm. Error bars denote standard deviation. Mann–Whitney *U* test was used for assessing statistical significance; ***p < 0.01. *Blebb.* blebbistatin, *EB* embryoid body

**Fig. 4:**
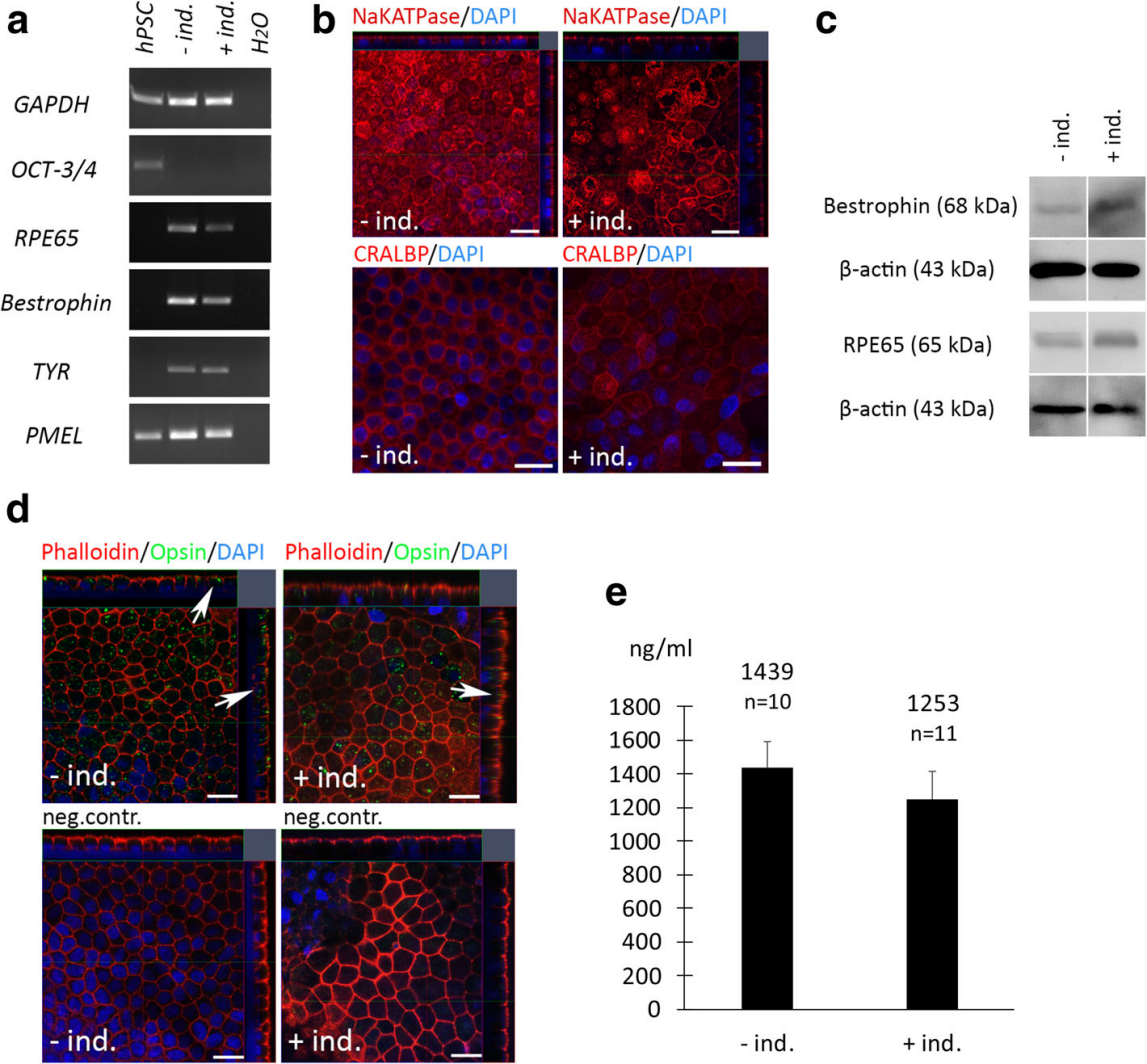
High-quality, functional RPE was obtained with the xeno-free differentiation method. **a** RT-PCR showing signature RPE gene expression, and **b** immunofluorescence labeling and **c** Western blot showing key RPE protein expression after final maturation on inserts for hESC1-RPE. Nuclei counterstained with DAPI; *scale bars* = 20 μm. **d** Confocal sections after 4 h POS feeding at +37 °C and at +4 °C (negative control; *neg.contr.*). *Arrows* indicate internalized POS labeled with anti-opsin antibody. *Scale bars* = 20 μm. **e** Mean PEDF secretion measured with ELISA from the apical side of cell culture inserts; *n* = number of inserts from two independent differentiation experiments. Error bars denote standard deviation. *+/– ind.* with and without induction, *hPSC* human pluripotent stem cell

After setting up and optimizing the feeder-free hPSC culture protocol, a RPE differentiation strategy was established with two representative hPSC lines (hESC1 and hiPSC1). Several approaches were tested to achieve a functional protocol (Additional file 6: Supplementary Dataset 1). Two-stage differentiation in 15% XF-Ko-SR medium, initiated in suspension (day 0–5), followed by adherent culture that was finalized on cell culture inserts, yielded mature RPE within a total of 19 weeks (Fig. 3a). Blebbistatin or ROCKi were required for viable EB formation, and a combination of LN-521 and col IV allowed for optimal attachment and RPE maturation. A 4-day neuroectodermal induction with the Wnt inhibitor IWP-2 and the transforming growth factor (TGF)-β inhibitor SB-505124 during the EB stage was used to direct differentiation. This neuroectodermal induction significantly (*p* < 0.01) increased the degree of pigmentation of the mature RPE layer (Fig. 3b and c) in a cell line-independent manner and repeatedly throughout seven separate differentiation experiments (five with hESC1 and two with hiPSC1). Spontaneous differentiation yielded smaller and more lightly pigmented RPE cells with a smoother epithelial morphology and significantly higher (*p* < 0.01) TEER values (Fig. 3d). The higher TEER indicates tight junction integrity and an intact epithelial barrier function. Moreover, zonula occludens-1 (ZO-1) and Claudin-19 (Cl-19) localization at junctional complexes indicated mature tight junctions, and their expression was similarly detected in cells differentiated with and without neuroectodermal induction (Fig. 3e). Confocal stacks of tight junction protein labeling confirmed monolayer structures. Despite the differences in pigmentation and TEER, cells differentiated with and without neuroectodermal induction expressed the RPE-specific genes *RPE65*, *BEST*, tyrosinase (*TYR*) and melanocyte protein *PMEL*, while lacking expression of the pluripotency marker *OCT-4* (Fig. 4a). They expressed visual cycle proteins RPE65 and cellular retinaldehyde binding protein (CRALBP), as well as the transporter proteins bestrophin and apically localized sodium-potassium adenosine triphosphatase (Na^+^K^+^-ATPase) (Fig. 4b and C). Furthermore, the cells showed correct RPE functionality by phagocytosis of porcine POS and apical secretion of high concentrations of PEDF (Fig. 4d and e). Finally, RPE cells differentiated with and without neuroectodermal induction showed normal karyotypes after 9 weeks of final maturation on cell culture inserts (data not shown). The characterization of hiPSC-RPE is shown in Additional file 7 (Figure S4). Overall, high-quality, functional RPE was derived using a completely xeno-free differentiation method from xeno- and feeder-free starting material, with 4-day neuroectodermal induction increasing the level of pigmentation of mature epithelial cell layers.

### Fine-tuning the early small molecule induction during two-stage, xeno-free differentiation guided hPSCs toward surface ectoderm and corneal LESC fate

**Fig. 5:**
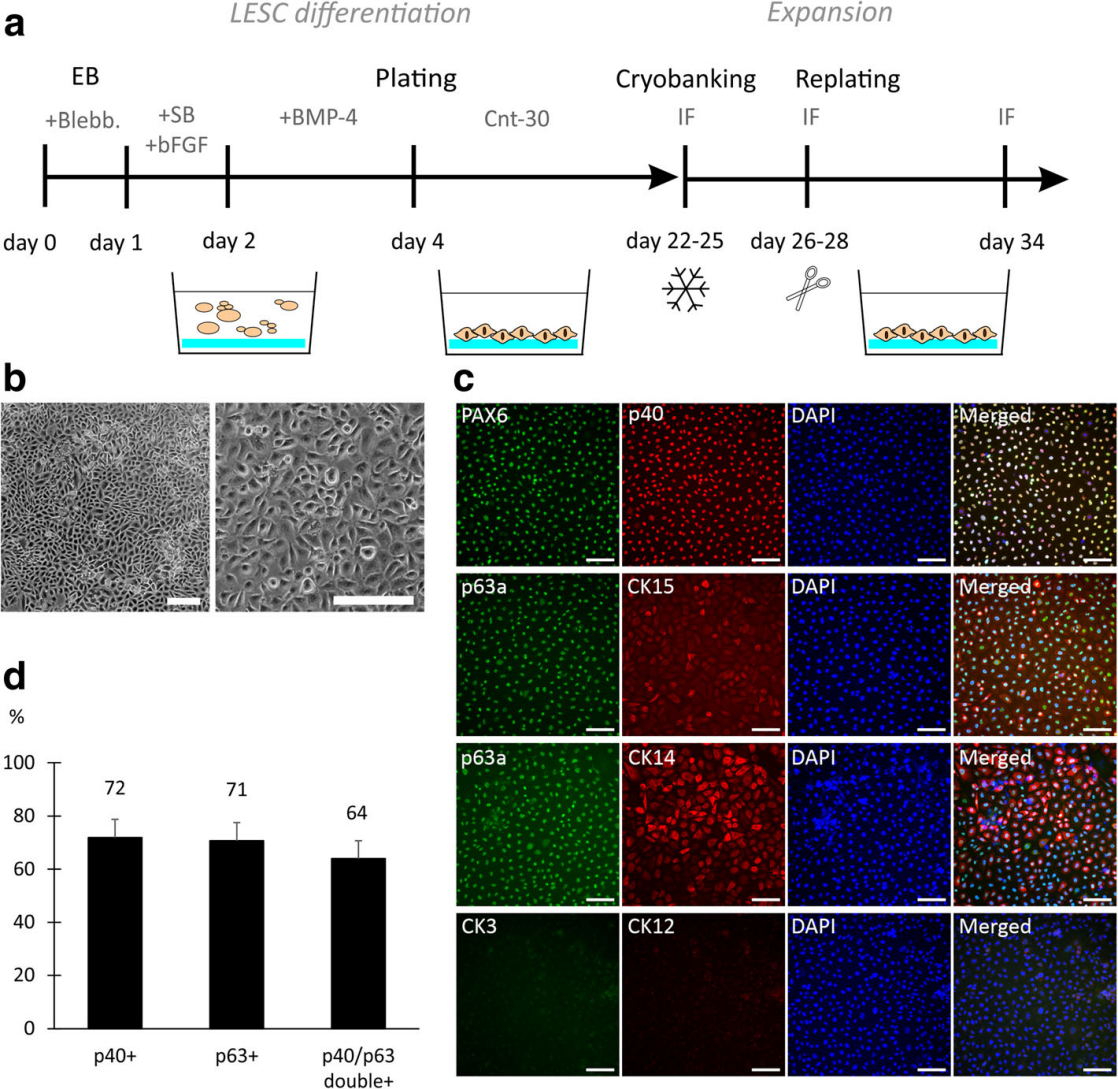
Directed differentiation of feeder-free hPSCs led to rapid hPSC-LESC production. **a** Schematic illustration of the differentiation strategy. **b** Phase-contrast images showing cell morphology after 22 days of differentiation for hESC1-LESCs; *scale bars* = 100 μm. **c** Expression of LESC markers after 22 days of differentiation. Nuclei counterstained with DAPI; *scale bars* = 100 μm. **d** Percentage of p40- and p63α-positive cells quantified from cytospin samples after 24 days of differentiation; *n* = 10 images, 1986 cells. *bFGF* basic fibroblast growth factor, *Blebb.* blebbistatin, *BMP* bone morphogenetic protein, *IF* immunofluorescence, *SB* SB-505124 hydrochloride hydrate

Next, we established a differentiation protocol to derive LESCs from two xeno- and feeder-free hPSC lines (hESC1 and hiPSC2). The same platform of 15% XF-Ko-SR-containing medium and the LN-521 and col IV combination matrix served as a starting point, and blebbistatin was used to promote aggregation and cell survival during EB formation. Our previously published protocol based on blocking TGF-β and Wnt signaling and activating FGF signaling, was established using hPSCs maintained on hFF feeder cells, and resulted in excessive cell death or neuronal differentiation of feeder-free hPSCs. Several strategies and modifications were tested to overcome this issue (see Additional file 8: Supplementary Dataset 2 and Additional file 9: Figure S5). Adding a mesodermal BMP4 induction after a short ectodermal induction directed differentiation toward surface ectoderm and corneal fate. Two-day induction with BMP4 after an overnight ectodermal induction with SB-505124 and bFGF, followed by adherent culture on LN-521 and col IV combination matrix in Cnt-30 epithelial medium, led to efficient differentiation of LESC-like epithelial cells (Fig. 5a). Already after 22 days of differentiation, the cells showed epithelial morphology (Fig. 5b), and expressed acknowledged LESC markers p40, p63α, and PAX6. Putative LESC markers cytokeratin-15 (CK15) and cytokeratin-14 (CK14) were also expressed in part, but mature corneal cytokeratins-12 (CK12) and -3 (CK3) were undetected at this stage (Fig. 5c). We observed a similar expression pattern using hiPSC (shown for hiPSC2-LESCs in Additional file 10: Figure S6). Cell counting confirmed 72% of the cells as p40 positive and 71% as p63α positive (and 64% double-positive for p40 and p63α) as early as day 24 of differentiation (Fig. 5d).

### Cryopreservation and successful recovery of hPSC-RPE cells and hPSC-LESCs enables generation of readily available cell products

**Fig. 6:**
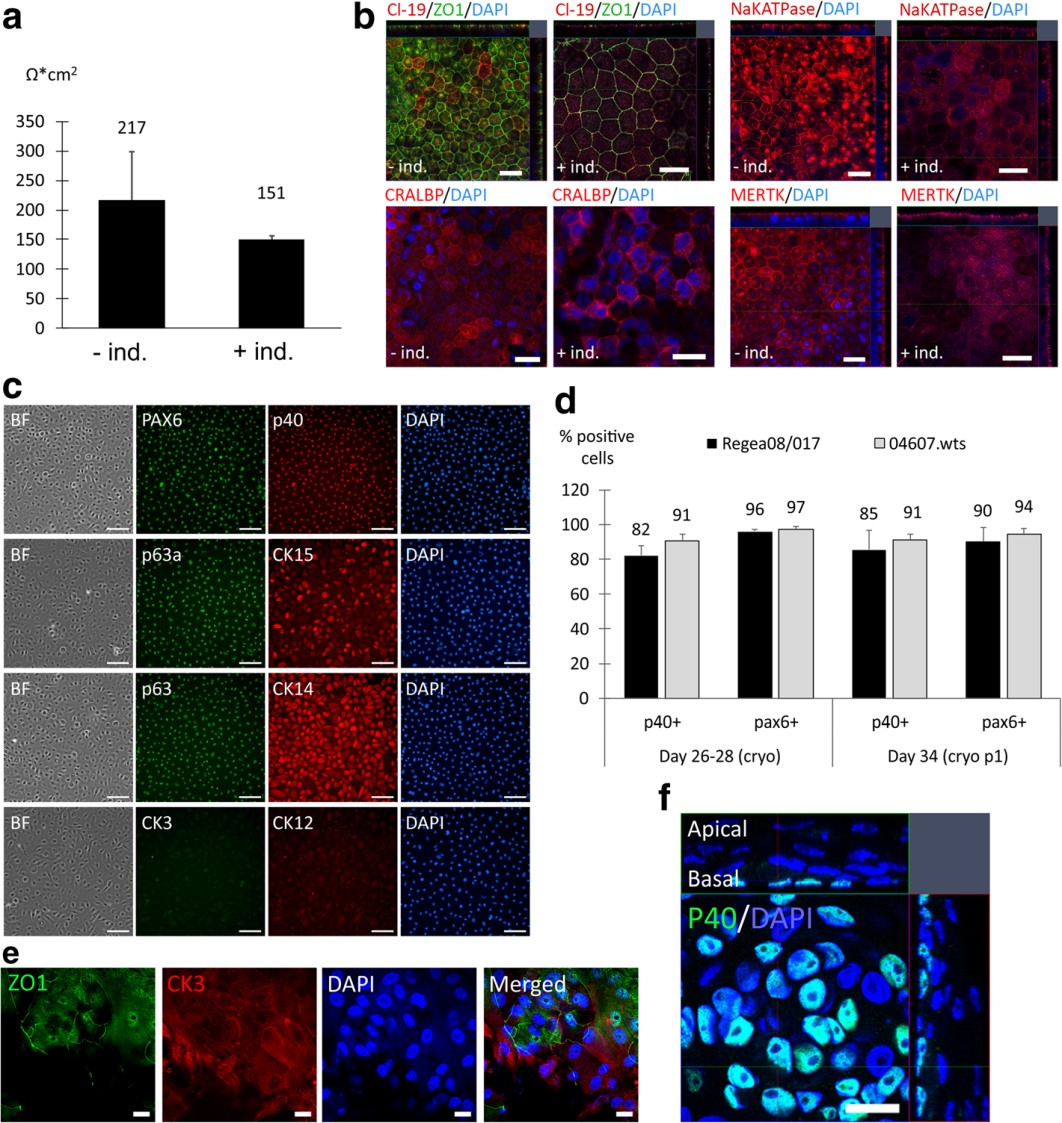
Human PSC-RPE and hPSC-LESCs maintained their cellular phenotypes after cryopreservation. **a** Human ESC1-RPE were cryopreserved for 52 days (*–ind.*) or for 281 days (*+ind.*) and thawed to 1.8 μg/cm^2^ LN-521 + 10 μg/cm^2^ col IV combination matrix. After 74 days (–ind.) and 71 days (+ind.) of post-thaw culture on inserts the cells showed (**a**) mean TEER values of 217 and 151 Ω*cm^2^ (*n* = 4 inserts and 6 inserts), (**b**) expression and localization of zonula occludens-1 (*ZO-1*) and Claudin-19 (*Cl-19*) to tight junctions, expression of the visual cycle protein cellular retinaldehyde binding prot (*CRALBP*), and apical expression of Na^+^K^+^ATPase and tyrosine-protein kinase Mer (*MERTK*); *scale bars* = 20 μm. **c** Human ESC1-LESCs, 4 days after recovery from cryostocks (103 days frozen, day 28 of differentiation in total) maintained morphology and expression of LESC markers similar to fresh cells; *scale bars* = 100 μm. A similar morphology and expression pattern was achieved after replating for expansion (day 34 in total). **d** Quantification of p40 and PAX6 expression for both hESC-LESCs (hESC1) and hiPSC-LESCs (hiPSC2) confirmed that the high LESC marker expression was retained during subsequent culture and replating after cryopreservation; *n* = 10 images, minimum of 2205 individual cells per time point. **e** After in vitro stratification assay, hPSC-LESCs showed expression of ZO1 tight junction protein and CK3, indicating maturity. **f** Confocal stacks confirmed expression of p40 at the basal layer of the stratified structure after 3 days of airlifting (shown for hiPSC2-LESCs). *Scale bars* = 20 μm. *BF* bright field

Cryobanking and recovery protocols for hPSC-derived RPE and LESCs were established using 40% XF-Ko-SR to buffer 10% DMSO medium, and cells were successfully recovered onto a LN-521 and col IV combination matrix. For hPSC-RPE, passaging was required to achieve an exponential growth phase for cryopreservation. The optimal window for cryostorage was 5–14 days after passaging, as RPE was changing morphology from fibroblastic to epithelial. The cryostoring was performed either after selection and plating (RPE passage level p1) or after additional replating to purify the RPE population (RPE passage level p2). Although we did not aim for maximal production speed or quantity, in practice we were able to cryostore an average of 2.2 × 10^6^ hESC1-RPE cells from 1.0 × 10^6^ pluripotent hESC1 cells in 64–84 days without induction (SD 2.3, *n* = 4 differentiation experiments), producing at best 5.6 × 10^6^ RPE from 1.0 × 10^6^ pluripotent hESC1 cells in 63 days. With induction we have been able to cryostore 1.04 × 10^6^ RPE cells from 1.0 × 10^6^ hESC1 cells as fast as in 42 days. After freezing at RPE p1, the cells required an additional passage for purification, allowing further expansion, while if frozen at RPE p2, the cells were directly thawed to cell culture inserts for final analysis. After recovery, hESC1 RPE cells were maintained on cell culture inserts for 10 weeks. They showed lower TEER compared to cells that had not gone through a cryopreservation step (Fig. 6a) but preserved their RPE marker expression status (Fig. 6b).

Human PSC-LESCs were cryostored at day 22–25 of differentiation when correct morphology and marker expression were detected. On average 0.72 × 10^6^ hESC1-LESC cells were produced for cryostorage from 1 × 10^6^ feeder-free hESC1 cells (SD 0.54, *n* = 6 differentiation experiments) by day 23 of differentiation. Human PSC-LESCs possessed appropriate epithelial morphology and LESC marker expression directly after recovery (4 days post-thawing, day 24–28 in total), and also after additional replating for expansion (day 34 in total) (Fig. 6c and Additional file 10: Figure S6). Over 80% of the cells expressed p40 and over 90% expressed PAX6 after cryostorage (Fig. 6d), suggesting that cryostoring and replating steps helped to purify the hPSC-LESC population even further. Moreover, the cells showed the capacity to form a stratified epithelium with apical tight junctions and expression of CK3 (Fig. 6e) as well as a p40-expressing stem cell population at the basal layer (Fig. 6f). Overall, successful cryopreservation protocols were established for RPE and LESCs, enabling cell banking that is essential for generation of future cell therapy products.

## Discussion

Clinical safety of cell-based therapies requires the use of highly defined and preferably xenogeneic-free techniques with economically reasonable cost. In this study, we developed simple but efficient methods for high-quality hPSC culture and differentiation towards RPE and LESC lineages. A minor modification of the inductive cues during the first week of differentiation steered hPSCs toward either the RPE or LESC fate, enabling production of two clinically relevant ocular cell types with the same basal method.

The culture of hPSCs has advanced from mouse or human feeder cell-based systems to feeder-independent culture on ECM proteins or their fragments in chemically defined, xeno-free media such as NutriStem® hESC XF medium (Biological Industries) and TeSR™2 medium [37]. The E8 medium was a major breakthrough as an albumin-free, current Good Manufacturing Practice (cGMP)-compliant medium containing only eight components essential for hPSC culture [38]. Consistent with earlier studies, we were able to achieve efficient monolayer culture of eight genetically distinct hESC and hiPSC lines in E8 medium combined with LN-521 matrix [39–41]. We found that prolonged feeder-free culture using single cell passaging led to karyotype changes that generated growth and differentiation advantages. In our analysis, two hPSC lines acquired karyotypic changes in the 20q arm—a known hot spot for chromosomal changes in hPSCs [42]. The gain of 20q11.21 allows hPSCs to escape apoptosis, allowing abnormal cells to take over the culture [43]. Feeder-free cultures thus require frequent karyotyping and use of low-passage hPSCs for differentiation. The safe window for usage is short; a maximum of 15 feeder-free passages equals 7.5 weeks of culture, while we have routinely cultured hPSCs on hFF feeders using manual passaging for up to 100 weeks with stable karyotypes. However, the feeder-free monolayer culture on LN-521 is more efficient, scalable, and less laborious compared to feeder-based systems. In practice, an average of approximately threefold cell multiplication pace was achieved within each passage (3–4 days) during successful culture. This means that a single 24-well of hPSCs (~100,000 cells) can expand into ~2.7 × 10^6^ cells within 3 passages (1.5 weeks). This efficiency enables the use of low-passage feeder-free hPSCs to produce clinically relevant numbers of differentiated cell types. Minimized variability due to culture medium and matrix simplicity, and flexibility as a weekend-free feeding regimen, also add value and decrease costs.

Besides the well-defined and high-quality hPSCs, successful stem cell-based therapy requires differentiation methods with sufficient yield and purity. Recently, several hPSC differentiation strategies mimicking whole eye development have been reported to generate multiple ocular cell types in zones of two-dimensional colonies [44], or complex three-dimensional corneal [13, 45] or retinal organoids [29]. Ocular organoids recapitulate the early developmental events in vitro and provide powerful three-dimensional model systems for studying developmental or disease processes. However, these protocols are complex and time-consuming, difficult to standardize for production of high cell yields for cell therapy applications, and would require rigorous sorting of the desired cell populations [46].

Within the last 2 years, several research groups introduced xeno-free protocols for spontaneous or directed clinical grade RPE differentiation [24–27, 29]. In our hands, attempts to replicate some of these methods were unsuccessful. Plaza Reyes et al. used spontaneous RPE differentiation in suspension in tailored NutriStem hESC XF medium lacking bFGF, and further plating of selected, pigmented RPE cells to LN-521 [25]. Our attempts to achieve RPE differentiation in EB suspensions led to disaggregation of EBs and insufficient levels of pigmentation. On the other hand, differentiation in X-VIVO™ 10 medium (Lonza), previously reported for xeno-free RPE differentiation [24], led to extreme pigmentation at the expense of RPE and epithelial properties (Additional file 6: Supplementary Dataset 1). RPE differentiation with XF-Ko-SR basal medium was recently reported by Choudhary and co-workers as an adherent monolayer-based method relying on temporal and sequential dual SMAD inhibition, BMP4, and Activin A activation [26]. Their approach was efficient and fast, with RPE-specific genes upregulated in 40 days, allowing whole plate passaging without the need for manual selection of pigmented cells. However, they found lower expression levels of key RPE genes in these cells compared with spontaneously differentiated hPSC-RPE. This again emphasizes the need for a prolonged culture of several weeks followed by purification and expansion to allow for proper RPE maturation. Our approach of using the XF-Ko-SR, together with LN-521 and col IV combination matrix, was suitable for either spontaneous or simple, directed, xeno-free RPE differentiation producing high-quality RPE. We also demonstrate that a similar method, with minor modifications, yields corneal LESCs; the induction stage was performed in XF-Ko-SR, and adherent culture was continued in CnT-30, a commercially available, fully defined culture medium developed for primary corneal epithelial cells. In its current formulation, CnT-30 contains animal-derived (but clinical grade) heparin, which could be replaced for a fully xeno-free medium. Most previously published studies to generate corneal epithelial cells from hPSCs have relied on the use of undefined, xenogeneic factors such as conditioned medium, PA6 feeder cells, Bowman’s, or amniotic membranes [16, 19, 47, 48]. A recent, defined protocol to differentiate a single hESC line to corneal epithelial progenitor cells utilized simple keratinocyte serum-free medium (KSFM) mixed with DMEM/F12 for increased calcium concentration, together with a simple col IV matrix [30]. ABCG2- and p63-positive populations were produced in just 6 days but differentiation required elevated CO_2_ levels. The mechanism behind these differentiation conditions was not discussed [30].

When directing feeder-free hPSC differentiation towards ocular epithelial lineages, suspension culture as EBs greatly improved cell survival, while small molecule induction helped direct differentiation. For feeder-free LESC differentiation, ectodermal induction alone was insufficient, resulting in neural network formation or cell death. A mesodermal induction with BMP4 was beneficial in directing differentiation towards surface ectoderm, and consequently LESCs. BMP4 is important in anterior eye segment development, induces corneal and epidermal differentiation, and inhibits neural differentiation [49, 50]. In our study, this two-stage surface ectodermal induction followed by adherent culture on LN-521/col IV combination matrix in CnT-30 medium resulted in > 70% p63α-positive cell populations in 24 days. As for RPE, the TGF-β signaling inhibitor SB-431542 has been previously shown to enhance pigmentation rate during initial differentiation, but in a cell line-dependent manner [51]. Meanwhile, Wnt signaling induces RPE differentiation once the cells are committed to the retinal fate [52, 53]. Our short neuroectodermal induction with Wnt and TGF-β inhibitors repeatedly increased pigmentation of mature RPE layers in all seven experiments and with both cell lines. The role of pigmentation resulting from melanin content in the RPE remains somewhat speculative. The pigment serves to store Ca^2+^, absorb stray light, and minimize scatter within the eye, but melanosomes also act as antioxidants, eliciting cytoprotective functions [54]. We have previously shown the inverse relationship of TEER and pigmentation [36], and continuously see this effect with hPSC-derived RPE. The mechanism behind this phenomenon and its clinical significance requires further studies. It was recently shown that the level of pigmentation does not correlate with hPSC-RPE maturity, although it is commonly used as such a marker [55]. Interestingly, highly pigmented hPSC-RPE appear to have significantly higher expression of pathways related to calcium signaling and adherens junction remodeling, compared with lightly pigmented cells [55]. Pigmentation may also affect other aspects of physiology and functionality of the intact RPE monolayer, and its relevance for transplantation success calls for further studies.

Ocular cell replacement therapies require relatively small populations of RPE (1.3 × 3.0 mm patch or 0.2 × 10^6^ cells in suspension) [11, 12] or a few thousand p63-bright LESCs [56] per eye, but quality assurance for GMP compliance requires additional cell populations and thus scale-up of culture and differentiation. RPE differentiation from hPSCs tends to be time consuming, so using fresh hPSC-RPE for cell replacement therapy requires a long waiting time for the patient. In contrast, the hPSC-LESC differentiation period is too rapid to allow for quality and safety testing prior to transplantation. Cryobanking offers a solution by allowing preparation of readily available cell stocks in advance. A recent study showed successful cryostoring and recovery of xeno-free hiPSC-derived neuroretinal organoids, and further isolated photoreceptor precursor cells, as well as RPE cells [29]. With our xeno-free methods, a starting culture of 1 × 10^6^ feeder-free hPSCs can yield cryostocks of > 5 × 10^6^ RPE cells in 9 weeks, and nearly 1 × 10^6^ LESCs within 23 days. Controlled passaging of hPSC-RPE once or twice is essential to achieve purity and successful cryostoring at the exponential growth phase, while passaging more than three times changes the hPSC-RPE phenotype [57]. Meanwhile, LESC purity improved after cryostoring and passaging, as suggested by ubiquitous p40 and p63α protein expression.

GMP-compliant derivation, culture, and differentiation methods are a requisite for cell-based medicinal products, including hPSC-derived cells [58]. Xeno-free, GMP-quality, clinical-grade hPSC-RPE products such as OpRegen® cells are emerging [28], but simplified protocols and raw materials for cell culture and differentiation are an advantage. The simple xeno-free methods described here could be upgraded to GMP-quality for future preclinical testing.

## Conclusions

We provide a simple but efficient methodology to produce two clinically relevant ocular epithelial cell types from feeder- and xeno-free hPSCs. Our results show that the simple, cost-effective methods produce high-quality RPE and LESCs from multiple cell lines and across several differentiation experiments. The safety and functional efficacy testing of the hPSC-RPE and hPSC-LESCs produced with these protocols are currently ongoing in non-human primates and rabbit models of LSCD, respectively.

## Additional files

Additional file 1: Supplementary materials and methods. (DOCX 65 kb)

Additional file 2: Figure S1. Human iPSC line hiPSC1 cultured on LN-521 in E8 medium maintained pluripotent characteristics. A) Typical undifferentiated morphology and B) expression of pluripotency markers NANOG, OCT-3/4, SSEA-3, SSEA-4, TRA-1-60, and TRA-1-81, as well as corresponding nuclear stains with DAPI after 4 passages in feeder-free culture. C) Pluripotency shown as expression of markers of the three embryonic germ layers, namely SOX17 for endoderm, OTX2 for ectoderm, and SMA for mesoderm after spontaneous differentiation (feeder-free passage level 4). All scale bars = 200 μm. D) Cells showing normal female karyotype after nine passages. (TIF 5463 kb)

Additional file 3: Figure S2. Human iPSC line hiPSC2 cultured on LN-521 in E8 medium maintained pluripotent characteristics. A) Typical undifferentiated morphology and B) expression of pluripotency markers OCT-3/4, SSEA-3, SSEA-4, TRA-1-60, and TRA-1-81, and LIN-28 as well as corresponding nuclear stains with DAPI after 11 passages in feeder-free culture. C) Pluripotency shown as expression of markers of the three embryonic germ layers after spontaneous differentiation (feeder-free passage level 8). All scale bars = 200 μm, except for SMA = 100 μm. D) Cells showing normal female karyotype after nine passages. (TIF 5580 kb)

Additional file 4: Figure S3. Human ESC line hESC1 cultured on VTN-N matrix in E8 medium using cluster passaging showed typical, distinct colony pattern, morphology, and expression of pluripotency markers. Bright field (BF) images after eight passages. The arrow pointing out some differentiating cells. Positive expression of pluripotency markers NANOG, OCT-3/4, SSEA-3, TRA-1-81, and TRA-1-60, and lack of expression of differentiation marker SSEA-1 after 14 passages in the feeder-free culture on VTN-N in E8 medium. Counterstaining of nuclei with DAPI. Scale bars = 200 μm. (TIF 1809 kb)

Additional file 5: Table S1. (DOCX 19 kb)

Additional file 6: Supplementary dataset 1. Optimization of hPSC-RPE differentiation. (DOCX 36 kb)

Additional file 7: Figure S4. High-quality, functional RPE was derived from the hiPSC1 line. A) Initial pigmentation rate was not affected by early neuroectodermal induction as shown in images of pigmented patches after 38 days of differentiation (upper row) with and without initial neuroectodermal induction (+/– ind.). Neuroectodermal induction increased pigmentation of the mature hiPSC1-RPE layer after 9 weeks of final culture on inserts, as shown in DIC confocal images (lower row). Scale bars = 10 μm. B) Similarly, the difference in pigmentation was reflected in the lower TEER of the highly pigmented RPE differentiated with induction, while very high TEER was achieved for the less pigmented cells after spontaneous differentiation; *n* = 6 inserts. C) RT-PCR showing signature RPE gene expression and lack of expression of the pluripotency marker OCT-3/4. D) IF labeling showing RPE protein expression and localization in vertical confocal sections for the junctional protein ZO-1, the transporter protein Na^+^K^+^-ATPase, and phagocytosis regulator protein tyrosine-protein kinase Mer (MERTK). E) Confocal sections after 4 h POS feeding at +37 °C and at +4 °C (negative control). Arrows indicate internalized POS labeled with anti-opsin antibody. Nuclei counterstained with DAPI; scale bars = 20 μm. F) Mean PEDF secretion measured with ELISA from the apical side of cell culture inserts; *n* = number of inserts. Error bars denote standard deviation. In addition, the hiPSC1-RPE cells showed normal karyotypes after differentiation with and without neuroectodermal induction (data not shown). (TIF 18107 kb)

Additional file 8: Supplementary dataset 2. Optimization of hPSC-LESC differentiation. (DOCX 42 kb)

Additional file 9: Figure S5. Feeder-free hPSCs responded differently to ectodermal induction compared to hPSCs cultured on feeder cells. Representative images of hESC1 after corneal differentiation with 10 μM SB-505124, 10 μM IWP-2, and 50 ng/ml FGF: A) neuronal networks (day 29), B) undesired cell morphology (day 21), and C) massive cell death (day 14) were observed; scale bars = 100 μm. Relative qPCR analysis of the early corneal differentiation from D) hFF feeder based culture, and E) feeder-free culture. Expression levels of undifferentiated hESCs (UD, blue bars) from the same culture system were used as a calibrator (fold-change value set to 1) for each gene. Relative fold-change values after embryoid body formation (EB, red bars) and after 4 or 6 days of induction (green bars). Error bars denote standard deviation. (TIF 7012 kb)

Additional file 10: Figure S6. Human iPSC-LESCs derived from the hiPSC2 line showed epithelial morphology and LESC marker expression at A) day 22 of differentiation, B) day 26 (in total) after cryopreservation (4 days post-thaw, 112 days frozen), and C) day 34 (in total) after cryopreservation and additional replating. Cells showed epithelial morphology in phase contrast (A) and bright field (BF) images, and expression of LESC markers PAX6, p40, p63α, cytokeratins 15 (CK15), and 14 (CK14), but no expression of mature corneal cytokeratins 12 and 3. Scale bars = 100 μm for all images. (TIF 7742 kb)

## Abbreviations

AMD: Age-related macular degeneration
bFGF: Basic fibroblast growth factor
BMP: Bone morphogenetic protein
BRB: Blood-retinal barrier
cGMP: Current Good Manufacturing Practice
CK: Cytokeratin
col IV: collagen type IV
CRALBP: cellular retinaldehyde binding protein
DMSO: Dimethyl sulfoxide
E8: Essential 8™ Flex Medium
EB: Embryoid body
ECM: Extracellular matrix
ELISA: Enzyme-linked immunosorbent assay
FF: Feeder-free
hESC: Human embryonic stem cell
hFF: human foreskin fibroblast
hiPSC: Human induced pluripotent stem cell
hPSC: Human pluripotent stem cell
Ko-SR: KnockOut™ Serum Replacement
LESC: limbal epithelial stem cell
LN-521: Recombinant laminin-521
LSCD: Limbal stem cell deficiency
OCT: octamer-binding transcription factor
PAX-6: Paired box protein Pax-6
PCR: Polymerase chain reaction
PEDF: Pigment epithelium-derived factor
PET: Polyethylene terephthalate
POS: Photoreceptor outer segments
ROCKi: Rock inhibitor Y-27632 dihydrochloride
RPE: Retinal pigment epithelial
RT-PCR: Reverse transcription polymerase chain reaction
SSEA: Stage-specific embryonic antigen
TEER: Transepithelial electrical resistance
TGF: transforming growth factor
VTN-N: Truncated human recombinant vitronectin
XF-ko-SR: KnockOut™ SR XenoFree CTS™

## References

[1] Dua HS, Shanmuganathan VA, Powell-Richards AO, Tighe PJ, Joseph A (2005). Limbal epithelial crypts: a novel anatomical structure and a putative limbal stem cell niche. Br J Ophthalmol.

[2] Yazdanpanah G, Jabbehdari S, Djalilian AR (2017). Limbal and corneal epithelial homeostasis. Curr Opin Ophthalmol.

[3] Strauss O (2005). The retinal pigment epithelium in visual function. Physiol Rev.

[4] Schmidt-Erfurth U, Chong V, Loewenstein A, Larsen M, Souied E, Schlingemann R (2014). Guidelines for the management of neovascular age-related macular degeneration by the European Society of Retina Specialists (EURETINA). Br J Ophthalmol.

[5] Notara M, Alatza A, Gilfillan J, Harris AR, Levis HJ, Schrader S (2010). In sickness and in health: corneal epithelial stem cell biology, pathology and therapy. Exp Eye Res.

[6] Osei-Bempong C, Figueiredo FC, Lako M (2013). The limbal epithelium of the eye—a review of limbal stem cell biology, disease and treatment. Bioessays.

[7] Ahmad S (2012). Concise review: limbal stem cell deficiency, dysfunction, and distress. Stem Cells Transl Med.

[8] Haagdorens M, Van Acker SI, Van Gerwen V, Ní Dhubhghaill S, Koppen C, Tassignon M (2016). Limbal stem cell deficiency: current treatment options and emerging therapies. Stem Cells Int.

[9] Atallah MR, Palioura S, Perez VL, Amescua G (2016). Limbal stem cell transplantation: current perspectives. Clin Ophthalmol.

[10] Miyagishima KJ, Wan Q, Miller SS, Bharti K. A basis for comparison: sensitive authentication of stem cell derived RPE using physiological responses of intact RPE monolayers. Stem Cell Transl Investig. 2017;4:e1497. PMC5341611 28286868

[11] Schwartz SD, Regillo CD, Lam BL, Eliott D, Rosenfeld PJ, Gregori NZ (2015). Human embryonic stem cell-derived retinal pigment epithelium in patients with age-related macular degeneration and Stargardt's macular dystrophy: follow-up of two open-label phase 1/2 studies. Lancet.

[12] Mandai M, Watanabe A, Kurimoto Y, Hirami Y, Morinaga C, Daimon T (2017). Autologous induced stem-cell-derived retinal cells for macular degeneration. N Engl J Med.

[13] Susaimanickam PJ, Maddileti S, Kumar V, Boyinpally SR, Naik RR, Naik MN, et al. Generating minicorneal organoids from human induced pluripotent stem cells. Development. 2017;144:2338–351. 10.1242/dev.143040 28559289

[14] Ali RR, Sowden JC (2011). Regenerative medicine: DIY eye. Nature.

[15] Klimanskaya I, Hipp J, Rezai KA, West M, Atala A, Lanza R (2004). Derivation and comparative assessment of retinal pigment epithelium from human embryonic stem cells using transcriptomics. Cloning Stem Cells.

[16] Ahmad S, Stewart R, Yung S, Kolli S, Armstrong L, Stojkovic M (2007). Differentiation of human embryonic stem cells into corneal epithelial-like cells by in vitro replication of the corneal epithelial stem cell niche. Stem Cells.

[17] Osakada F, Jin ZB, Hirami Y, Ikeda H, Danjyo T, Watanabe K (2009). In vitro differentiation of retinal cells from human pluripotent stem cells by small-molecule induction. J Cell Sci.

[18] Idelson M, Alper R, Obolensky A, Ben-Shushan E, Hemo I, Yachimovich-Cohen N (2009). Directed differentiation of human embryonic stem cells into functional retinal pigment epithelium cells. Cell Stem Cell.

[19] Hayashi R, Ishikawa Y, Ito M, Kageyama T, Takashiba K, Fujioka T (2012). Generation of corneal epithelial cells from induced pluripotent stem cells derived from human dermal fibroblast and corneal limbal epithelium. PLoS One.

[20] Buchholz DE, Pennington BO, Croze RH, Hinman CR, Coffey PJ, Clegg DO (2013). Rapid and efficient directed differentiation of human pluripotent stem cells into retinal pigmented epithelium. Stem Cells Transl Med.

[21] Mikhailova A, Ilmarinen T, Uusitalo H, Skottman H (2014). Small-molecule induction promotes corneal epithelial cell differentiation from human induced pluripotent stem cells. Stem Cell Reports.

[22] Sareen D, Saghizadeh M, Ornelas L, Winkler MA, Narwani K, Sahabian A (2014). Differentiation of human limbal-derived induced pluripotent stem cells into limbal-like epithelium. Stem Cells Transl Med.

[23] Maruotti J, Sripathi SR, Bharti K, Fuller J, Wahlin KJ, Ranganathan V (2015). Small-molecule-directed, efficient generation of retinal pigment epithelium from human pluripotent stem cells. Proc Natl Acad Sci U S A.

[24] Pennington BO, Clegg DO, Melkoumian ZK, Hikita ST (2015). Defined culture of human embryonic stem cells and xeno-free derivation of retinal pigmented epithelial cells on a novel, synthetic substrate. Stem Cells Transl Med.

[25] Plaza Reyes A, Petrus-Reurer S, Antonsson L, Stenfelt S, Bartuma H, Panula S (2016). Xeno-free and defined human embryonic stem cell-derived retinal pigment epithelial cells functionally integrate in a large-eyed preclinical model. Stem Cell Reports.

[26] Choudhary P, Booth H, Gutteridge A, Surmacz B, Louca I, Steer J (2017). Directing differentiation of pluripotent stem cells toward retinal pigment epithelium lineage. Stem Cells Transl Med.

[27] Geng Z, Walsh PJ, Truong V, Hill C, Ebeling M, Kapphahn RJ (2017). Generation of retinal pigmented epithelium from iPSCs derived from the conjunctiva of donors with and without age related macular degeneration. PLoS One.

[28] McGill TJ, Bohana-Kashtan O, Stoddard JW, Andrews MD, Pandit N, Rosenberg-Belmaker LR (2017). Long-term efficacy of GMP grade xeno-free hESC-derived RPE cells following transplantation. Transl Vis Sci Technol.

[29] Reichman S, Slembrouck A, Gagliardi G, Chaffiol A, Terray A, Nanteau C (2017). Generation of storable retinal organoids and retinal pigmented epithelium from adherent human iPS cells in xeno-free and feeder-free conditions. Stem Cells.

[30] Zhang C, Du L, Pang K, Wu X (2017). Differentiation of human embryonic stem cells into corneal epithelial progenitor cells under defined conditions. PLoS One.

[31] Skottman H (2010). Derivation and characterization of three new human embryonic stem cell lines in Finland. In Vitro Cell Dev Biol Anim.

[32] Ahola A, Kiviaho AL, Larsson K, Honkanen M, Aalto-Setälä K, Hyttinen J (2014). Video image-based analysis of single human induced pluripotent stem cell derived cardiomyocyte beating dynamics using digital image correlation. Biomed Eng Online.

[33] Ojala M, Prajapati C, Pölönen R, Rajala K, Pekkanen-Mattila M, Rasku J (2016). Mutation-specific phenotypes in hiPSC-derived cardiomyocytes carrying either myosin-binding protein C or α-tropomyosin mutation for hypertrophic cardiomyopathy. Stem Cells Int.

[34] Toivonen S, Ojala M, Hyysalo A, Ilmarinen T, Rajala K, Pekkanen-Mattila M (2013). Comparative analysis of targeted differentiation of human induced pluripotent stem cells (hiPSCs) and human embryonic stem cells reveals variability associated with incomplete transgene silencing in retrovirally derived hiPSC lines. Stem Cells Transl Med.

[35] Vaajasaari H, Ilmarinen T, Juuti-Uusitalo K, Rajala K, Onnela N, Narkilahti S (2011). Toward the defined and xeno-free differentiation of functional human pluripotent stem cell-derived retinal pigment epithelial cells. Mol Vis.

[36] Sorkio A, Hongisto H, Kaarniranta K, Uusitalo H, Juuti-Uusitalo K, Skottman H (2014). Structure and barrier properties of human embryonic stem cell-derived retinal pigment epithelial cells are affected by extracellular matrix protein coating. Tissue Eng Part A.

[37] Ludwig TE, Levenstein ME, Jones JM, Berggren WT, Mitchen ER, Frane JL (2006). Derivation of human embryonic stem cells in defined conditions. Nat Biotechnol.

[38] Chen G, Gulbranson DR, Hou Z, Bolin JM, Ruotti V, Probasco MD (2011). Chemically defined conditions for human iPSC derivation and culture. Nat Methods.

[39] Lu HF, Chai C, Lim TC, Leong MF, Lim JK, Gao S (2014). A defined xeno-free and feeder-free culture system for the derivation, expansion and direct differentiation of transgene-free patient-specific induced pluripotent stem cells. Biomaterials.

[40] Tano K, Yasuda S, Kuroda T, Saito H, Umezawa A, Sato Y (2014). A novel in vitro method for detecting undifferentiated human pluripotent stem cells as impurities in cell therapy products using a highly efficient culture system. PLoS One.

[41] Uhlin E, Rönnholm H, Day K, Kele M, Tammimies K, Bölte S (2017). Derivation of human iPS cell lines from monozygotic twins in defined and xeno free conditions. Stem Cell Res.

[42] Amps K, Andrews PW, Anyfantis G, Armstrong L, Avery S, International Stem Cell Initiative (2011). Screening ethnically diverse human embryonic stem cells identifies a chromosome 20 minimal amplicon conferring growth advantage. Nat Biotechnol.

[43] Avery S, Hirst AJ, Baker D, Lim CY, Alagaratnam S, Skotheim RI (2013). BCL-XL mediates the strong selective advantage of a 20q11.21 amplification commonly found in human embryonic stem cell cultures. Stem Cell Reports.

[44] Hayashi R, Ishikawa Y, Sasamoto Y, Katori R, Nomura N, Ichikawa T (2016). Co-ordinated ocular development from human iPS cells and recovery of corneal function. Nature.

[45] Foster JW, Wahlin K, Adams SM, Birk DE, Zack DJ, Chakravarti S (2017). Cornea organoids from human induced pluripotent stem cells. Sci Rep.

[46] Hayashi R, Ishikawa Y, Katori R, Sasamoto Y, Taniwaki Y, Takayanagi H (2017). Coordinated generation of multiple ocular-like cell lineages and fabrication of functional corneal epithelial cell sheets from human iPS cells. Nat Protoc.

[47] Hanson C, Hardarson T, Ellerström C, Nordberg M, Caisander G, Rao M (2013). Transplantation of human embryonic stem cells onto a partially wounded human cornea in vitro. Acta Ophthalmol.

[48] Zhu J, Zhang K, Sun Y, Gao X, Li Y, Chen Z (2013). Reconstruction of functional ocular surface by acellular porcine cornea matrix scaffold and limbal stem cells derived from human embryonic stem cells. Tissue Eng Part A.

[49] Wilson PA, Hemmati-Brivanlou A (1995). Induction of epidermis and inhibition of neural fate by BMP-4. Nature.

[50] Metallo CM, Ji L, de Pablo JJ, Palecek SP (2008). Retinoic acid and bone morphogenetic protein signaling synergize to efficiently direct epithelial differentiation of human embryonic stem cells. Stem Cells.

[51] Lane A, Philip LR, Ruban L, Fynes K, Smart M, Carr A (2014). Engineering efficient retinal pigment epithelium differentiation from human pluripotent stem cells. Stem Cells Transl Med.

[52] Westenskow P, Piccolo S, Fuhrmann S (2009). Beta-catenin controls differentiation of the retinal pigment epithelium in the mouse optic cup by regulating Mitf and Otx2 expression. Development.

[53] Fuhrmann S (2008). Wnt signaling in eye organogenesis. Organogenesis.

[54] Rózanowski B, Burke JM, Boulton ME, Sarna T, Rózanowska M (2008). Human RPE melanosomes protect from photosensitized and iron-mediated oxidation but become pro-oxidant in the presence of iron upon photodegradation. Invest Ophthalmol Vis Sci.

[55] Bennis A, Jacobs JG, Catsburg LaE, Ten Brink JB, Koster C, Schlingemann RO, et al. Stem cell derived retinal pigment epithelium: the role of pigmentation as maturation marker and gene expression profile comparison with human endogenous retinal pigment epithelium. Stem Cell Rev. 2017 10.1007/s12015-017-9754-0 PMC5602068 28730556

[56] Rama P, Matuska S, Paganoni G, Spinelli A, De Luca M, Pellegrini G (2010). Limbal stem-cell therapy and long-term corneal regeneration. N Engl J Med.

[57] Singh R, Phillips MJ, Kuai D, Meyer J, Martin JM, Smith MA (2013). Functional analysis of serially expanded human iPS cell-derived RPE cultures. Invest Ophthalmol Vis Sci.

[58] Salmikangas P, Menezes-Ferreira M, Reischl I, Tsiftsoglou A, Kyselovic J, Borg JJ (2015). Manufacturing, characterization and control of cell-based medicinal products: challenging paradigms toward commercial use. Regen Med.

