# Supplementary material for: Xeno- and feeder-free differentiation of human pluripotent stem cells to two distinct ocular epithelial cell types using simple modifications of one method
Source: Stem Cell Res Ther. 2017 Dec 29;8:291. doi: 10.1186/s13287-017-0738-4 (PMC5747074; doi:10.1186/s13287-017-0738-4)
Supplement: Supplementary file 1 — Supplementary materials and methods. (DOCX 65 kb) [file 13287_2017_738_MOESM1_ESM.docx]

**Supplementary Materials and Methods**

**Pluripotent Stem Cell Culture**

For feeder-free culture, hPSC colonies on hFF feeder layers were manually dissected and transferred onto Corning® CellBIND® 24-well culture plates coated with human recombinant laminin-521 (LN-521, Biolamina, Sweden). LN-521 coating was carried out as instructed by the manufacturer, diluted in 1xDPBS containing Ca^2+^ and Mg^2+^ (Gibco, Thermo Fisher Scientific), overnight at +4°C. The coating dilution was removed, but not rinsed, before cell seeding. LN-521 concentration of 1.09 µg/cm^2^ was used for the first passage and 0.55 µg/cm^2^ for subsequent passages. Essential 8™ Medium or Essential 8™ Flex Medium (E8, Thermo Fisher Scientific), was supplemented with 50x supplement and 50 U/ml Penicillin-Streptomycin (Gibco, Thermo Fisher Scientific), and used as recommended by the manufacturer, bringing to room temperature (RT) before introducing to cells. Single cell passaging with xeno-free 1X TrypLE™ Select Enzyme was done by 3-4 min incubation at +37°C, allowing the cells to round up but not detach, followed by enzyme removal and inactivation with Defined Trypsin Inhibitor (Gibco, Thermo Fisher Scientific). Cells were detached by pipetting, centrifuged to pellet, suspended to fresh media, counted, and replated in fresh medium. Cells were passaged twice a week at 80-100% confluency, using constant split ratio of 40 000-50 000 cells/cm^2^. Weekend-free feeding regimen was achieved by passaging on Thursdays and Mondays.

For feeder-free culture on truncated recombinant human vitronectin (VTN-N, Gibco, Thermo Fisher Scientific), hESC1 line and hiPSC3 line were transferred from hFF feeder cells as described above. Corning® CellBIND® 6-well culture plates were coated with 0.5 µg/cm^2^ VTN-N diluted in 1xDPBS without Ca^2+^ and Mg^2+^ (Gibco, Thermo Fisher Scientific) for 1 hour at RT, as instructed by manufacturer. Passaging was done at approximately 85% confluency by cluster passaging with UltraPure™ 0.5 M EDTA, pH 8.0 (Invitrogen, Thermo Fisher Scientific) as instructed by the supplier. EDTA was incubated on cells for 5-8 min at RT, removed from the cells as colonies began to round up, and cells dislodged to fresh E8 medium by gently pipetting with a 5 ml pipette. Cells were passaged every 4-6 days and if in excess, colonies with differentiated morphology were removed before passaging. A varying split ratio of 1:6 – 1:10 depending on the density of the colonies was used.

**Pluripotent Stem Cell Characterizations**

Human PSCs were continuously monitored for attachment, growth, and morphology with Nikon Eclipse TE2000-S phase contrast microscope (Nikon Instruments Europe B.V. Amstelveen, Netherlands).

***Immunofluorescence***

Immunofluorescence labeling for pluripotency markers were done as previously described [1] using the following antibodies: NANOG (1:200, R&D Systems, AF1997) OCT-3/4 (1:200, R&D Systems, AF1759), SSEA-3 (1:600, R&D Systems, MAB1434), SSEA-4 (1:200, R&D Systems, MAB1435), TRA-1-60 (1:200, Millipore, MAB4360), TRA-1-81 (1:200, Santa Cruz Biotechnology SC-21706), LIN-28 (1:400, ThermoFisher Scientific, MA1-016), and SSEA-1 (1:200, Santa Cruz Biotechnology, SC-21702). Appropriate Alexa Fluor 488- or 568-conjugated secondary antibodies: 1:800 donkey anti-goat IgG (A11055, A11057), goat anti-Mouse IgM (A21042), donkey anti-mouse Ig (A21202, A10037)(all from Thermo Fisher Scientific), and 1:400 FITC-conjugated Goat anti-Rat IgM (7102, Novus Biologicals) were used. Nuclei were counterstained with 4´, 6´ diamidino-2-phenylidole (DAPI) included in the VECTASHIELD™ Mounting Medium (Vector Laboratories Inc., Burlingame, CA). Images were acquired with Olympus IX51 phase contrast microscope with fluorescence optics and Olympus DP30BW camera (Olympus Corporation, Tokyo, Japan).

***Flow Cytometry***

Flow cytometry analysis was performed for hESC1 cultured for 12 passages on LN-521 in E8 medium and the same cell line cultured on hFF feeder cells in standard hESC medium for 46 passages. The colonies used for flowcytometry had an undifferentiated morphology. Cells were dissociated with TrypLE™ Select Enzyme and counted with a hemocytometer using trypan blue exclusion. For SSEA-4 labeling, 1x10^5^ cells per sample were washed twice with FACS buffer containing 0.5% BSA (Sigma-Aldrich) and 0.01% NaN_3_ (Sigma-Aldrich) in DPBS, and labeled with 8 µl PE-conjugated mouse anti-SSEA-4 (BD Biosciences, 560128) or PE-conjugated mouse IgG3 κ Isotype Control (BD Biosciences, 559926), for 30 min on ice. Cells for negative samples were treated similarly, but not labeled. The cells were washed twice and analyzed in FACS buffer. For OCT-3/4 labeling, the cells were fixed with 4% paraformaldehyde (PFA) for 10 min at RT, washed twice with SAP buffer containing 0.1% Saponin (Sigma-Aldrich) and 0.05% NaN_3_ in DPBS, labeled with 10 µl PE-conjugated mouse anti-OCT3/4 (Human Isoform A, BD Biosciences, 561556) or PE-conjugated mouse IgG κ Isotype Control (BD Biosciences, 554680), for 30 minutes at RT, washed and analyzed in SAP buffer. All samples were analyzed with BD Accuri C6 Flow Cytometer and software (BD Biosciences) with acquisition set to at least 10 000 events per sample and gating performed with the unlabeled sample.

***In Vitro Pluripotency Assay***

Human PSC pluripotency was verified by spontaneous differentiation as EBs, followed by immunofluorescence labeling for derivative cells of the three embryonic germ lineages. Cells were detached either with TrypLE™ Select Enzyme or by manual scraping, and transferred to Corning® Costar® Ultra-Low attachment plates in EB-medium: KnockOut™ DMEM supplemented with 10% fetal bovine serum (FBS, Gibco, Thermo Fisher Scientific), 2 mM GlutaMAX™ Supplement, 0.1 mM 2-Mercaptoethanol, 1% MEM Non-Essential Amino Acids Solution, and 50 U/ml Penicillin-Streptomycin. Blebbistatin (5-10 µM) was added for the first 24 h. EB-medium was changed the following day and three times a week there after. After about 14 days of differentiation, the EBs were plated onto VTN-N or LN-521 coated plates and cultured for 7 days. The cells were then washed, fixed with 4% PFA and labeled similarly to undifferentiated hPSCs as described in [1]. Following primary antibodies were used: alpha-smooth muscle actin (SMA, 1:400, R&D Systems, MAB1420) for mesoderm, alpha-fetoprotein (AFP, 1:200, R&D Systems MAB1369) or SOX17 (1:200, R&D Systems, AF1924) for endoderm, and nestin clone 10C2 (1:1000, Merck Millipore, MAB5326) or OTX2 (1:200, R&D Systems, AF1979) for ectoderm. Primary antibodies were omitted for secondary controls. Images were acquired with Olympus IX51 microscope.

***Karyotyping***

Karyotyping was performed at Finnish Microarray and Sequencing Centre (FMSC), Turku Centre for Biotechnology with the KaryoLite BoBs assay (Perkin Elmer).

**Differentiation to Ocular Cells**

Differentiation to ocular cells was performed by either manually detaching undifferentiated hPSCs with EDTA followed by gentle scraping with pipette tip, or enzymatically with TrypLE™ Select Enzyme. Differentiation was performed in XF-Ko-SR medium: KnockOut™ DMEM supplemented with 15% KnockOut™ SR XenoFree CTS™, 2 mM GlutaMAX™, 0.1 mM 2-Mercaptoethanol, 1% MEM Non-Essential Amino Acids Solution, and 50 U/ml Penicillin-Streptomycin (all from Gibco, Thermo Fisher Scientific). Cells were plated onto Corning® Costar® Ultra-Low attachment plates to induce embryoid body (EB) formation with 5 µM or 10 µM blebbistatin (Sigma-Aldrich) or Rock inhibitor Y-27632 dihydrochloride (R&D Systems), overnight at +37°C.

***RPE Differentiation***

The following day after EB formation, the medium was changed and neuroectodermal small molecule induction added to half of the cells (+ ind.) while the other half underwent spontaneous differentiation (- ind.). 10 µM SB-505124 hydrochloride hydrate (Sigma-Aldrich) and 10 µM Wnt Antagonist II, IWP-2 (Merck Millipore) were used for neuroectodermal induction. During the 3-6 day induction period, the medium was changed daily. At day 4-6 of differentiation the cells were plated down to Corning® CellBIND® 12-well culture plates coated with 0.75 µg/cm^2^ LN-521 and 10 µg/cm^2^ human placental collagen Type IV (col IV, Sigma-Aldrich), overnight at +4°C. The coating was removed, rinsed once with DPBS and EBs plated down in XF-Ko-SR medium. The media were thereafter changed three times a week and cells monitored for appearance of pigmentation. For the first passaging, pigmented foci were manually cut with scalpel, washed twice with DPBS, dissociated with 10-15 min incubation with TrypLE™ Select Enzyme, pelleted by centrifugation and plated down to new culture wells coated with LN-521 and col IV in fresh medium. For further passaging, the RPE cells were directly detached with TrypLE™ Select Enzyme as above and counted. For final passage, 250,000 RPE cells /cm^2^ were plated to similarly coated polyethylene terephthalate (PET) hanging cell culture insert with 1.0 µm pore size in a 24-well plate (Merck Millipore).

***LESC Differentiation***

The following day after EB formation, the medium was changed to XF-Ko-SR medium supplemented with 10 µM SB-505124 and 50 ng/ml human basic fibroblast growth factor (bFGF) (PeproTech Inc., Rocky Hill, NJ). For the following two days, the medium was changed to XF-Ko-SR medium supplemented with 25 ng/ml BMP-4 (PeproTech Inc.). After a total of 4 days of surface ectodermal induction in suspension culture, the EBs were plated onto Corning® CellBIND® 12-well culture plates coated with 0.75 µg/cm^2^ LN-521 and 5 µg/cm^2^ col IV in a defined and serum-free medium CnT-30 (CELLnTEC Advanced Cell Systems AG, Bern, Switzerland) with the density of approx. 50 EBs per well. The cells were thereafter cultured in CnT-30 medium that was changed three times a week.

**Characterization of hPSC-RPE Cells**

***Gene Expression Analysis***

RPE-specific gene expression was assessed with reverse transcription polymerase chain reaction (RT-PCR). Undifferentiated hPSC cultured on LN-521 in E8 medium served as control. Gene expression for retinal pigment epithelium specific protein 65 kDa (*RPE65*), bestrophin (*BEST*), tyrosinase (*TYR*), premelanosome protein (*PMEL*), and pluripotency marker octamer-binding transcription factor (*OCT-3/4*) was analyzed. Glyceraldehyde 3-phosphate dehydrogenase (*GAPDH*) was used as an endogenous control. RNA was extracted with Qiagen RNeasy Plus Mini kit (Qiagen, Valencia, CA, USA) according to manufacturer's instructions. The RNA concentration and quality were assessed with NanoDrop 1000 spectrophotometer (NanoDrop Technologies, Wilmington, DE, USA). PCR grade water was used as a negative control in RT-PCR analysis. Detailed protocols for cDNA reverse transcription, PCR as well as the primer sequences have been reported previously [2].

***Quantification of Pigmentation and size***

The degree of pigmentation and cell size were quantified from randomly-selected images captured of hESC1-RPE after 9 weeks of final culture on inserts for cells cultured without (–ind.) and with neuroectodermal induction (+ ind.). For quantifying pigmentation level, 20 images in total (from two independent differentiation experiments) were taken with AxioScope A1 fluorescence microscope (Carl Zeiss, Jena, Germany) using a 40x objective. The light exposure settings and illumination were maintained constant between the media. The intensity of the pigmentation was quantified with ImageJ Image Processing and Analysis software (ImageJ, https://imagej.nih.gov/ij/) through pixel intensity normalization. The background pixel intensity of the empty insert was subtracted from each sample set (+/- induction). Pigmentation intensity was presented relative to - ind. condition. Size difference quantifications were carried out from phalloidin, (Tetramethylrhodamine B isothiocyanate, 1:500 dilution, P1951 Sigma) -stained images acquired with LSM 700 Confocal microscope (Carl Zeiss, Jena, Germany) using 63× oil immersion objective. Five images with> 1000 cells in total were included for both – ind. and + ind. conditions. Analyze particles pluging of ImageJ was used. Images were converted to binary, and “Watershed” command was used to separate merged areas. A set of regions of interest (ROIs), representing cell areas, was automatically created based on binary values. Automatic ROI selections were then compared to original images and inaccurate selections were manually corrected.

***Immunofluorescence***

Immunofluorescence labeling for hPSC-RPE was done with the protocol described in [2], using the following primary antibodies: CRALBP (1:200, Abcam, ab15051), Claudin-19 (1:50, R&D Systems, MAB6970), ZO-1/TJP1 (1:100, 61-7300, Thermo Fisher Scientific), alpha 1 Sodium Potassium ATPase (1:200, Abcam, ab7671), and MERTK (1:50, Abnova, H00010461-M01). Alexa Fluor 568-conjugated goat anti-mouse IgG (A-11004) and 488-conjugated donkey anti-rabbit Ig (A-21206), both diluted 1:400, were used as secondary antibodies (both from Thermo Fisher Scientific). Phalloidin (1:500) was used for labeling filamentous actin. Nuclei were counterstained with DAPI included in the VECTASHIELD™ Mounting Medium or ProLong® Gold Antifade Mountant with DAPI (Thermo Fisher Scientific). Images were captured with LSM 700 Confocal microscope using a 40x objective or 63× oil immersion objective and edited using ZEN 2011 Black Edition (Carl Zeiss).

***Transepithelial Electrical Resistance***

Transepithelial electrical resistance (TEER) was measured with a Millicell electrical resistance system volt-ohm meter (Merck Millipore, Darmstad, Germany) from cell culture inserts without balancing to RT before measuring. TEER values (Ω/cm^2^) were calculated by subtracting the TEER value of empty inserts cultured in XF-Ko-SR medium in the absence of cells, and by multiplying by the surface area of the insert (0.3 cm^2^).

***Enzyme-Linked Immunosorbent Assay***

The capacity of RPE cells differentiated from hESC1 and hiPSC1 to secrete PEDF was studied with Enzyme-linked immunosorbent assay (ELISA). Apical culture media (400 µl) of several replicate cell culture inserts was collected after 19 h incubation at +37°C. The media samples were stored at -70°C until analyzed with Human PEDF ELISA kit (BioVendor, Brno,Czech Republic) as instructed by the manufacturer. Media samples incubated without cells were collected for background measurements. Samples were diluted 300x with dilution buffer and measured as duplicates. Optical densities were measured with Perkin Elmer Wallac 1420 VICTOR2™ Microplate Reader (Perkin Elmer-Wallace, Norton, OH, USA).

***In Vitro Phagocytosis Assay***

The functional capacity of the hPSC-RPE to phagocytose porcine photoreceptor outer segments (POS) was assessed *in vitro*. Porcine POS were isolated from porcine eyes, collected to cold AMES' medium (Sigma- Aldrich) with 0.5 % Penicillin-Streptomycin immediately after slaughter and kept on ice, protected from light until POS extraction approximately 2 h later. The retinas were removed using scissors, blades and tweezers in a dark room under red light. Retinas were homogenized with gentle shaking in 0.73 M sucrose phosphate buffer, filtered twice through a gauze and cell types separated in sucrose gradient (0.75 M, 1.0 M, 1.25 M, 1.5 M, 1.75 M) by ultracentrifugation (Optima ultracentrifuge, Beckman Coulter, Inc., Brea, CA) 112,400g for 48 min at +4°C. The faint pink POS layer was collected in phosphate buffer, washed twice with centrifugation 3000 g for 10 min at +4°C, and stored as aliquots in 73 mM sucrose phosphate buffer at -70°C. For phagocytosis assay, POS were defrosted, centrifuged 5400 rpm, 4 min (Eppendorf Centrifuge 5415 R) washed with DPBS, centrifuged again and suspended into the medium containing 10% FBS. POS were added to the apical side of the hPSC-RPE inserts and incubated protected from light for 4 h at +37°C, or at +4°C for negative controls. The inserts were washed several times with DPBS and fixed with 4% PFA for 15 min at RT followed by DPBS washes. Cells were permeabilized with 0.1% Triton-X (Sigma-Aldrich) in DPBS for 15 min at RT, followed by DPBS washes, and blocked with 3% BSA (Sigma-Aldrich) in DPBS for 1 hour at RT, followed by DPBS washes. The cells were then labeled with monoclonal anti-opsin antibody (Sigma 04886), diluted 1:200 in 0.5 % BSA, in PBS overnight at +4°C. After DPBS washes the cells were labeled with 1:400 dilution of Alexa Fluor 488-conjugated donkey anti-mouse IgG (A-21202) and 1:500 Phalloidin in 0.5 % BSA in DPBS for 1 hour at RT. After several DPBS washes the cells were mounted with VECTASHIELD™ Mounting Medium containing DAPI. Z-stack images were acquired with confocal microscope to visualize internalized POS (LSM 700, Carl Zeiss, 63× oil immersion objective).

***Western Blot***

RPE65, and Bestrophin protein expression levels were studied with Western blotting with protocol previously described in [3]. Primary antibodies 1:5000 Anti-RPE65 (MAB5428, Merck Millipore) and 1:1000 Anti-Bestrophin (ab14928, Abcam) were used. After stripping the membranes, 1:2000 β-Actin Antibody (sc-47778, Santa Cruz Biotechnology) was used as loading control. 1:3000 HRP-conjugated goat anti-mouse IgG (sc-2005, Santa Cruz) and 1:2000 HRP-conjugated polyclonal swine anti-rabbit IgG (P0217 Dako) were used as secondary antibodies.

**Characterization of hPSC-LESC Cells**

***Gene Expression Analysis***

Relative gene expression during early LESC differentiation was assessed with quantitative PCR (qPCR) analysis. Relative expression of *OCT-3/4*, *PAX6*, ATP-binding cassette sub-family G member 2 (*ABCG2*), *BMP-4*, and pituitary homeobox 2 (*PITX2*) was studied during 7-day induction with 10 µM SB-505124, 10 µM IWP-2, and 50 ng/ml bFGF for hESC1 cultured on both hFF feeder cells in standard hESC medium and for cells cultured on LN-521 in E8 medium. For cells cultured on hFF, samples were collected from undifferentiated (UD) cells, overnight blebbistatin treatment to form EBs, and on day 7, after six days of treatment with induction medium. For feeder-free hPSCs, the samples were collected from UD cells, after 72 h blebbistatin treatment, and on day 7, after four days of treatment with induction medium. For RNA extraction, cDNA synthesis, and sequence-specific TaqMan Gene Expression Assays (Applied Biosystems), refer to [4]. All samples and controls were run as triplicate reactions with the 7300 Real-Time PCR system and results analyzed with the 7300 System SDS Software (Applied Biosystems) based on the cycle threshold (CT) values. Relative quantification of each gene was calculated by applying the -2^∆∆Ct^ method. Results were normalized to *GAPDH* expression with the undifferentiated hESCs as the calibrator (set as one) for both culture methods separately.

***Immunofluorescence***

The hPSC-LESC cells were characterized with immunofluorescence labeling after 24 days of differentiation, and after recovery from cryopreservation (day 26-28 and day 34). Immunofluorescence labeling for the hPSC-LESC was conducted as described in [4]. The following primary antibodies and dilutions were used: rabbit anti-PAX6 (1:200, Sigma-Aldrich, HPA030775), mouse anti-p40 (1:100, BioCare Medical, ACI3066A), p63α (1:200,Cell Signaling Technology, 4892), cytokeratin 15 Ab-1 (1:200, Thermo Fisher Scientific, MS-1068-P), cytokeratin 14 (1:300, R&D Systems, MAB3164), anti-cytokeratin 3 [AE5] (1:200, Abcam, ab77869) and cytokeratin 12 [L-20] (1:200, SantaCruz, sc-17099). Alexa Fluor 568-conjugated donkey anti-goat Ig (A-11057) and donkey anti-mouse Ig (A-10037) as well as Alexa Fluor 488-conjugated donkey anti-rabbit Ig (A-21206) and donkey anti-mouse Ig (A-21202), all diluted at 1:800, were used as secondary antibodies (all from Thermo Fisher Scientific). Samples were mounted with VECTASHIELD™ Mounting Medium with DAPI, and images were acquired with Olympus IX51 microscope.

***Quantification of p40, p63α, and PAX6 Expression***

Quantification of p40 and p63α was performed from double-stained cytospin samples labeled with respective antibodies at the time point of day 24. Cytospin samples were prepared as described in [4]. For cell counting, 10 images in total were captured with 10x magnification from randomly selected locations in two separate slides. Quantification of p40 and PAX6 was performed from samples fixed and double-stained with respective antibodies directly in the culture vessels at the time points of 26-28 and 34 days. For cell counting, 10 images in total were captured with 10x magnification from the middle and each cardinal point area of two wells. Cells were counted with ImageJ Image Processing and Analysis software tools. Region of interests (ROIs) were highlighted by thresholding and thereafter grayscale image was converted to binary. Binary processing tools “Dilate”, “Fill holes” and “Watershed” were selectively used to promote more accurate selection. Lastly, each set of ROIs was evaluated against the original grayscale image and inaccurate selections were manually corrected. For each image, percentages of p40 or p63α and PAX6 or p40 positively stained nuclei were calculated against DAPI stained nuclei. For the cytospin samples, 1986 individual cells were analyzed. For quantification of p40 and PAX6, a minimum of 2205 individual cells per time point were analyzed.

***In Vitro* Stratification Assay**

Stratification of hPSC-LESC derived epithelium was carried out with cryopreserved hiPSC2-LESC. The cells were thawed onto ⌀10 mm transparent LinkCell-100 membrane (LinkoCare Life Sciences Ltd, Sweden) in CnT-30 medium supplemented with 50 U/ml Penicillin-Streptomycin (Gibco, Thermo Fisher Scientific), in the density of 100 000 cells per membrane. Stratification was induced from confluent cultures by using 0.1 mM calcium chloride supplementation (Lonza) for one day and air-liquid interface culturing for three subsequent days, or alternatively without air-lifting by co-culturing hPSC-LESC indirectly with 3T3 mouse embryonic fibroblast feeder cells in 1% fetal bovine serum (FBS, Gibco) and 0.1 mM calcium chloride enriched CnT-30 media for nine days. During air-lifting media was changed daily, otherwise three times a week. The membranes were then washed with DPBS and fixed with 4% PFA for 20 min at RT, followed by DPBS washes. Immunofluorescence labeling against mouse anti-p40 and rabbit anti-ZO1 (1:100, Invitrogen, 61-7300) antibodies were carried out as for characterization of hPSC-LESC, but using ProLong® Gold Antifade with DAPI as mounting medium. Z-stack images of the samples were acquired with confocal microscope to visualize the layered structure and tight junctions of the formed epithelia (LSM 700, Carl Zeiss, 63× oil immersion objective).

**Statistical Analysis and Software**

Statistical significance was tested using the non-parametric Mann–Whitney U-test in IBM SPSS Statistics software. P-values <0.05 were considered statistically significant. Bar charts were created with Microsoft Excel 2016, and Adobe Photoshop CC 2017 and CorelDRAW X7 were used to edit and create images.

**References**

1. Hakala H, Rajala K, Ojala M, Panula S, Areva S, Kellomäki M, et al. Comparison of biomaterials and extracellular matrices as a culture platform for multiple, independently derived human embryonic stem cell lines. Tissue Eng Part A 2009;15:1775-85.

2. Vaajasaari H, Ilmarinen T, Juuti-Uusitalo K, Rajala K, Onnela N, Narkilahti S, et al. Toward the defined and xeno-free differentiation of functional human pluripotent stem cell-derived retinal pigment epithelial cells. Mol. Vis. 2011;17:558-75.

3. Sorkio A, Hongisto H, Kaarniranta K, Uusitalo H, Juuti-Uusitalo K, Skottman H. Structure and barrier properties of human embryonic stem cell-derived retinal pigment epithelial cells are affected by extracellular matrix protein coating. Tissue Eng Part A. 2014;20(3-4):622-34.

4. Mikhailova A, Ilmarinen T, Uusitalo H, Skottman H. Small-molecule induction promotes corneal epithelial cell differentiation from human induced pluripotent stem cells. Stem Cell Reports. 2014;2(2):219-31.
