# Supplementary figures and images for: Xeno- and feeder-free differentiation of human pluripotent stem cells to two distinct ocular epithelial cell types using simple modifications of one method
Source: Stem Cell Res Ther. 2017 Dec 29;8:291. doi: 10.1186/s13287-017-0738-4 (PMC5747074; doi:10.1186/s13287-017-0738-4)

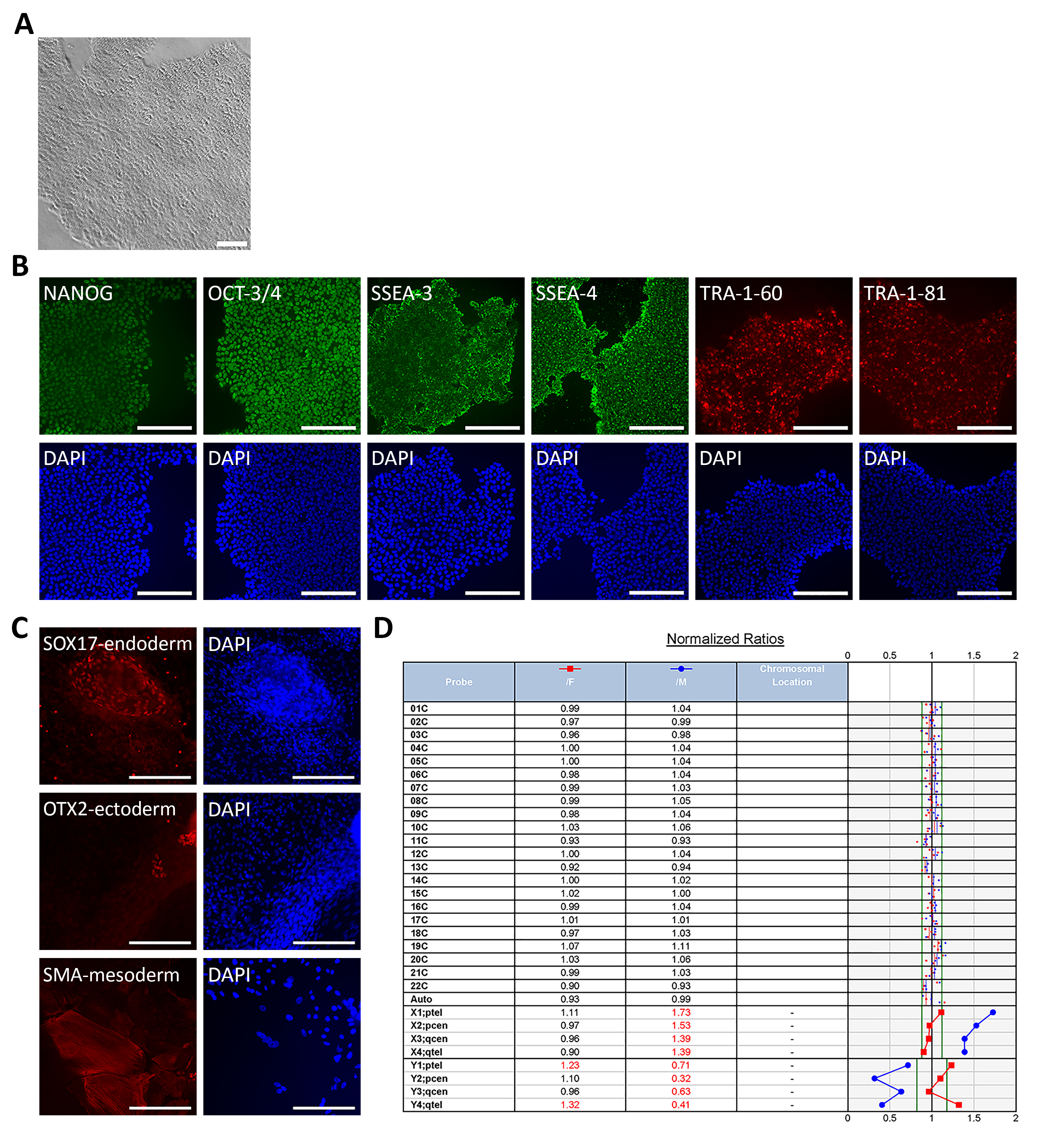

Supplement: Supplementary file 2 — Human iPSC line hiPSC1 cultured on LN-521 in E8 medium maintained pluripotent characteristics. A) Typical undifferentiated morphology and B) expression of pluripotency markers NANOG, OCT-3/4, SSEA-3, SSEA-4, TRA-1-60, and TRA-1-81, as well as corresponding nuclear stains with DAPI after 4 passages in feeder-free culture. C) Pluripotency shown as expression of markers of the three embryonic germ layers, namely SOX17 for endoderm, OTX2 for ectoderm, and SMA for mesoderm after spontaneous differentiation (feeder-free passage level 4). All scale bars = 200 μm. D) Cells showing normal female karyotype after nine passages. (TIF 5463 kb) [file 13287_2017_738_MOESM2_ESM.tif]

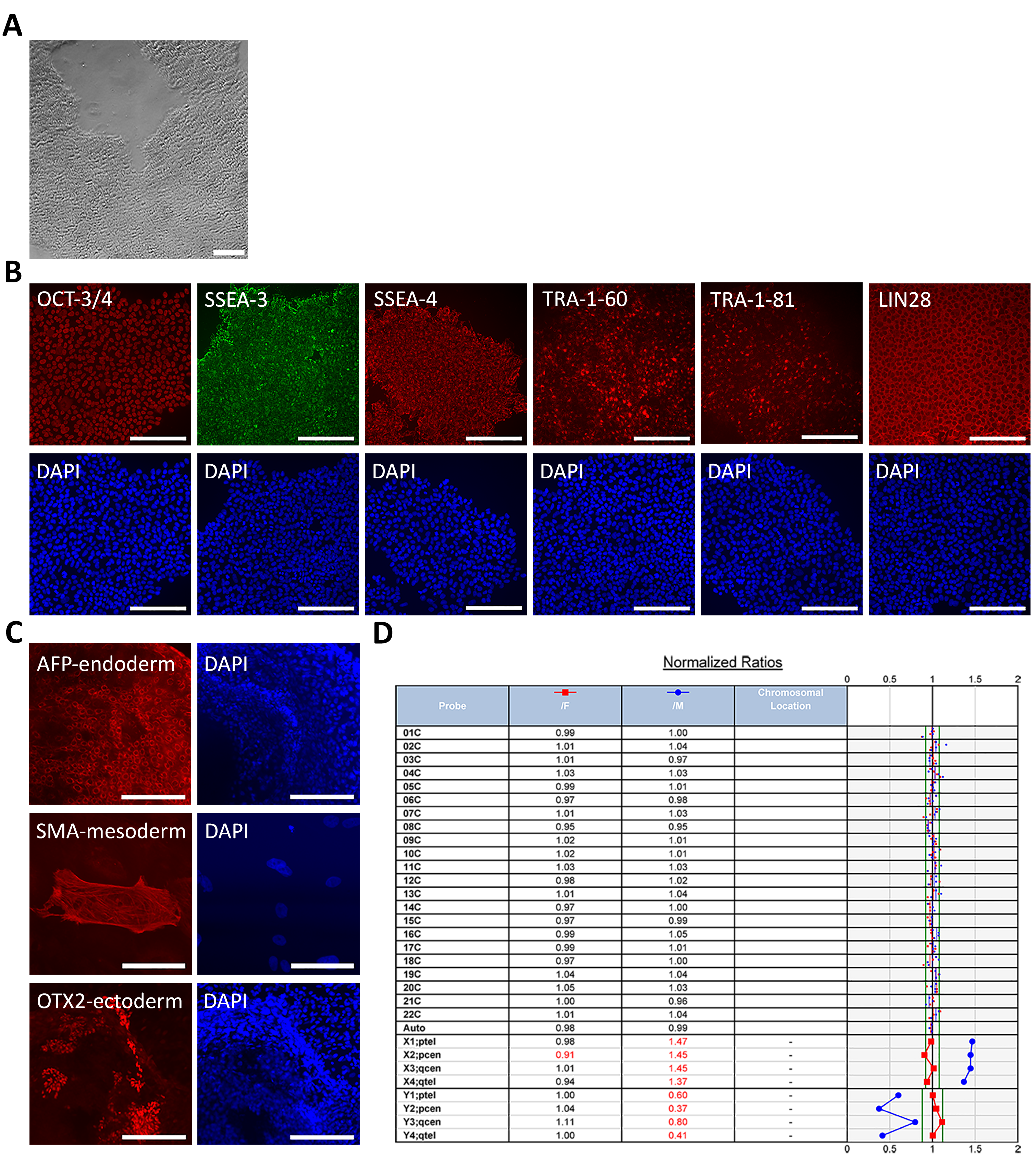

Supplement: Supplementary file 3 — Human iPSC line hiPSC2 cultured on LN-521 in E8 medium maintained pluripotent characteristics. A) Typical undifferentiated morphology and B) expression of pluripotency markers OCT-3/4, SSEA-3, SSEA-4, TRA-1-60, and TRA-1-81, and LIN-28 as well as corresponding nuclear stains with DAPI after 11 passages in feeder-free culture. C) Pluripotency shown as expression of markers of the three embryonic germ layers after spontaneous differentiation (feeder-free passage level 8). All scale bars = 200 μm, except for SMA = 100 μm. D) Cells showing normal female karyotype after nine passages. (TIF 5580 kb) [file 13287_2017_738_MOESM3_ESM.tif]

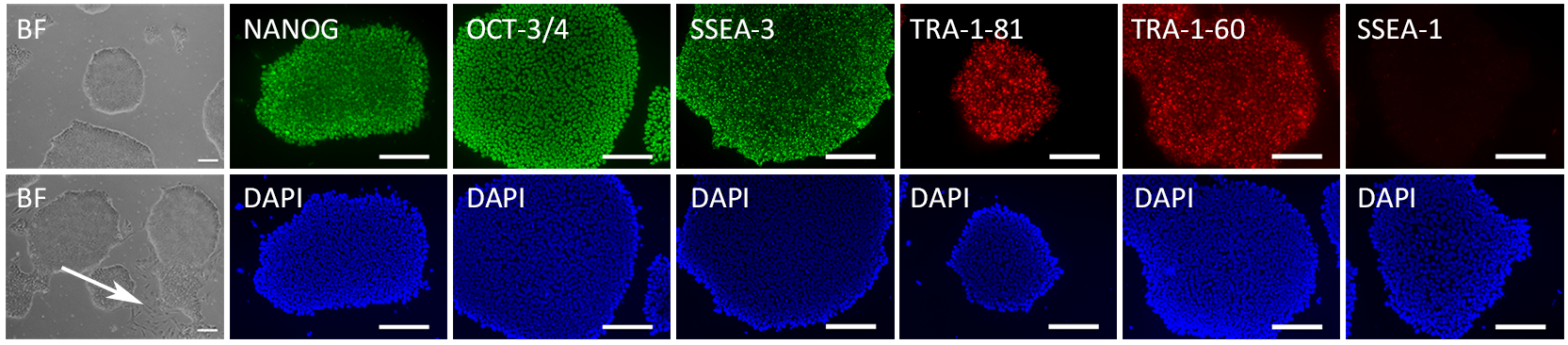

Supplement: Supplementary file 4 — Human ESC line hESC1 cultured on VTN-N matrix in E8 medium using cluster passaging showed typical, distinct colony pattern, morphology, and expression of pluripotency markers. Bright field (BF) images after eight passages. The arrow pointing out some differentiating cells. Positive expression of pluripotency markers NANOG, OCT-3/4, SSEA-3, TRA-1-81, and TRA-1-60, and lack of expression of differentiation marker SSEA-1 after 14 passages in the feeder-free culture on VTN-N in E8 medium. Counterstaining of nuclei with DAPI. Scale bars = 200 μm. (TIF 1809 kb) [file 13287_2017_738_MOESM4_ESM.tif]

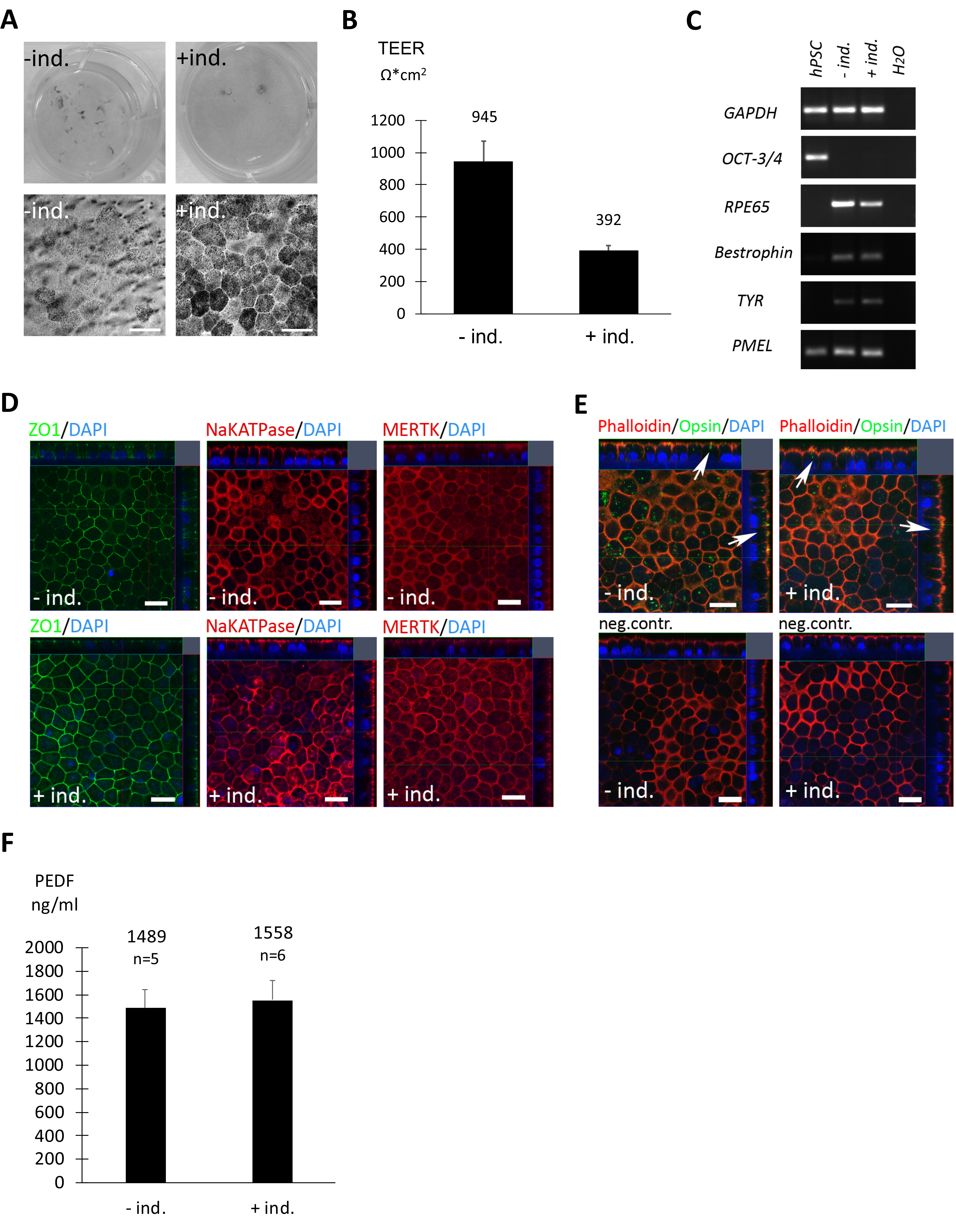

Supplement: Supplementary file 7 — High-quality, functional RPE was derived from the hiPSC1 line. A) Initial pigmentation rate was not affected by early neuroectodermal induction as shown in images of pigmented patches after 38 days of differentiation (upper row) with and without initial neuroectodermal induction (+/– ind.). Neuroectodermal induction increased pigmentation of the mature hiPSC1-RPE layer after 9 weeks of final culture on inserts, as shown in DIC confocal images (lower row). Scale bars = 10 μm. B) Similarly, the difference in pigmentation was reflected in the lower TEER of the highly pigmented RPE differentiated with induction, while very high TEER was achieved for the less pigmented cells after spontaneous differentiation; n = 6 inserts. C) RT-PCR showing signature RPE gene expression and lack of expression of the pluripotency marker OCT-3/4. D) IF labeling showing RPE protein expression and localization in vertical confocal sections for the junctional protein ZO-1, the transporter protein Na+K+-ATPase, and phagocytosis regulator protein tyrosine-protein kinase Mer (MERTK). E) Confocal sections after 4 h POS feeding at +37 °C and at +4 °C (negative control). Arrows indicate internalized POS labeled with anti-opsin antibody. Nuclei counterstained with DAPI; scale bars = 20 μm. F) Mean PEDF secretion measured with ELISA from the apical side of cell culture inserts; n = number of inserts. Error bars denote standard deviation. In addition, the hiPSC1-RPE cells showed normal karyotypes after differentiation with and without neuroectodermal induction (data not shown). (TIF 18107 kb) [file 13287_2017_738_MOESM7_ESM.tif]

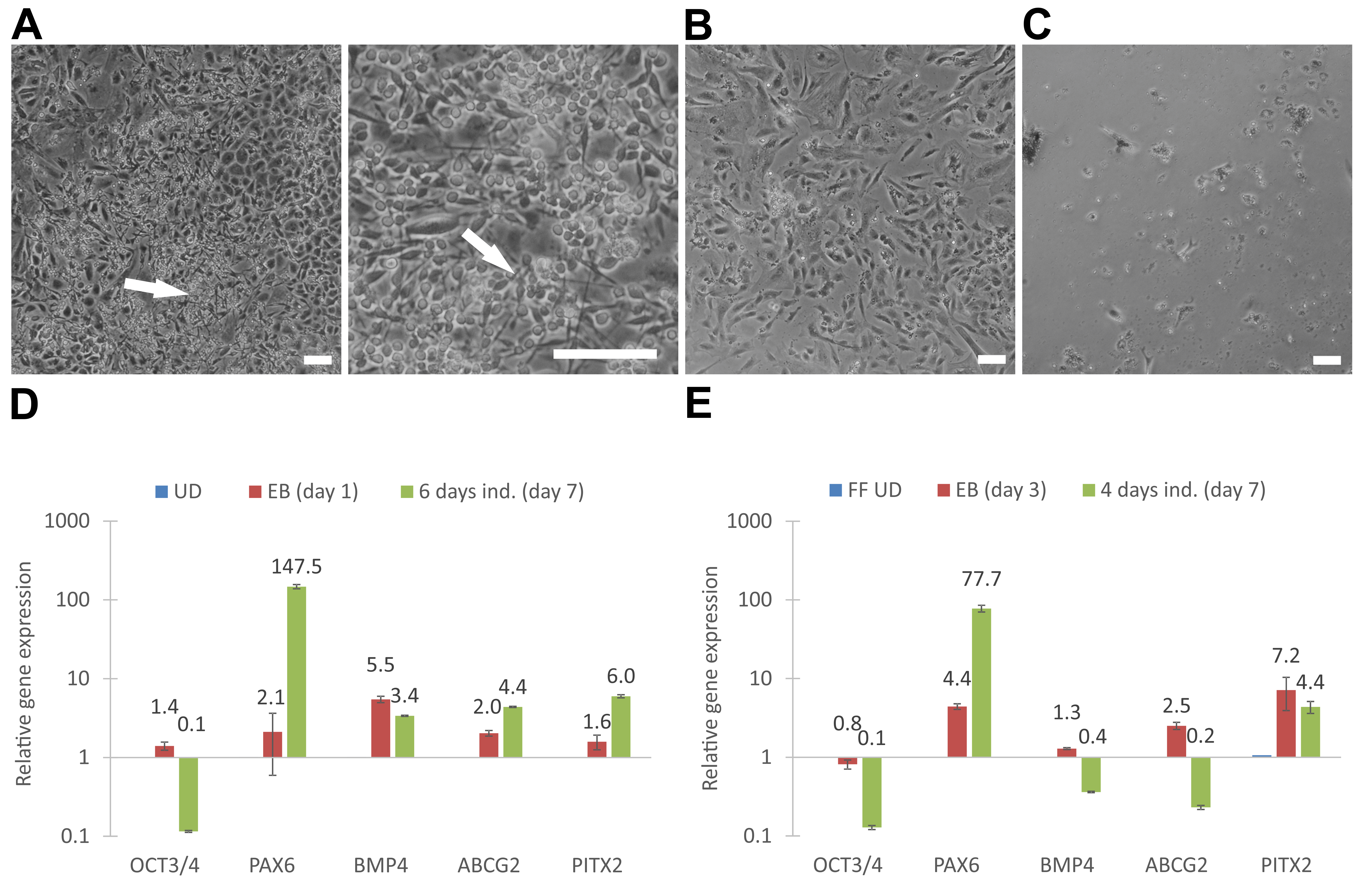

Supplement: Supplementary file 9 — Feeder-free hPSCs responded differently to ectodermal induction compared to hPSCs cultured on feeder cells. Representative images of hESC1 after corneal differentiation with 10 μM SB-505124, 10 μM IWP-2, and 50 ng/ml FGF: A) neuronal networks (day 29), B) undesired cell morphology (day 21), and C) massive cell death (day 14) were observed; scale bars = 100 μm. Relative qPCR analysis of the early corneal differentiation from D) hFF feeder based culture, and E) feeder-free culture. Expression levels of undifferentiated hESCs (UD, blue bars) from the same culture system were used as a calibrator (fold-change value set to 1) for each gene. Relative fold-change values after embryoid body formation (EB, red bars) and after 4 or 6 days of induction (green bars). Error bars denote standard deviation. (TIF 7012 kb) [file 13287_2017_738_MOESM9_ESM.tif]

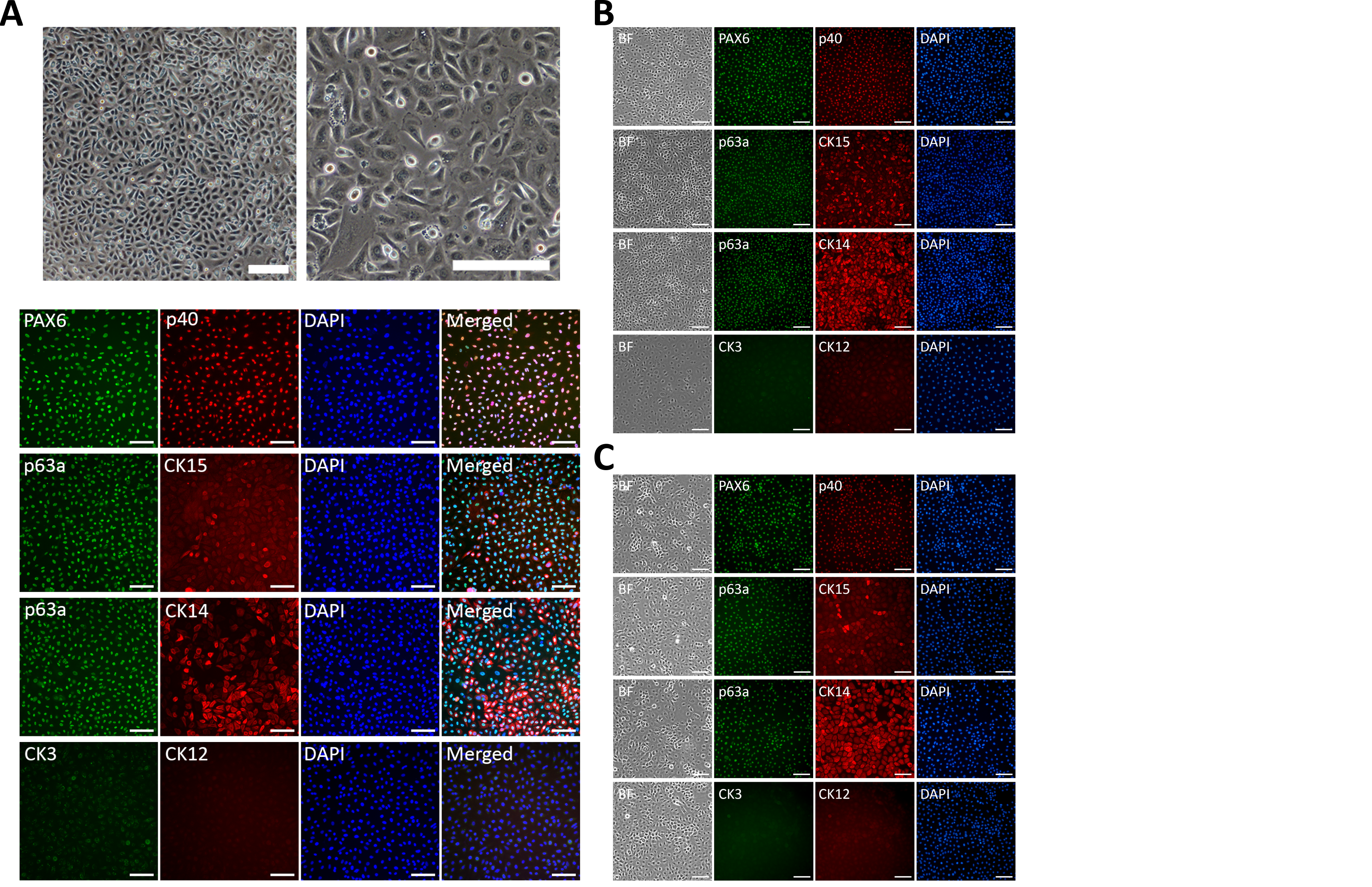

Supplement: Supplementary file 10 — Human iPSC-LESCs derived from the hiPSC2 line showed epithelial morphology and LESC marker expression at A) day 22 of differentiation, B) day 26 (in total) after cryopreservation (4 days post-thaw, 112 days frozen), and C) day 34 (in total) after cryopreservation and additional replating. Cells showed epithelial morphology in phase contrast (A) and bright field (BF) images, and expression of LESC markers PAX6, p40, p63α, cytokeratins 15 (CK15), and 14 (CK14), but no expression of mature corneal cytokeratins 12 and 3. Scale bars = 100 μm for all images. (TIF 7742 kb) [file 13287_2017_738_MOESM10_ESM.tif]
