# Supplementary material for: Xeno- and feeder-free differentiation of human pluripotent stem cells to two distinct ocular epithelial cell types using simple modifications of one method
Source: Stem Cell Res Ther. 2017 Dec 29;8:291. doi: 10.1186/s13287-017-0738-4 (PMC5747074; doi:10.1186/s13287-017-0738-4)
Supplement: Supplementary file 5 — (DOCX 19 kb) [file 13287_2017_738_MOESM5_ESM.docx]

**Supplementary Table S1.** hPSC multiplication capacity and doubling times within each passage (cells passaged twice a week) on LN-521 in E8 medium for hESC1 and hiPSC2 cell lines. Total number of cells plated (cells initial) and cells harvested at the end of passage (cells final) used for calculations shown. Doubling time calculated with formula DT=time*log10(2)/log10(cells initial)-log10(cells final).

| hESC1 |  |  |  |  |  |
| --- | --- | --- | --- | --- | --- |
| Passage  (Feeder-free passage) | **Cells**  **initial** | **Cells**  **final** | **Time**  **(hours)** | **Multiplication within passage** | **Doubling Time. DT (hours)** |
| p25(3FF) | 1 200 000 | 3 200 000 | 72 | 2.67 | 50.88 |
| p26(4FF) | 900 000 | 2 450 000 | 96 | 2.72 | 66.45 |
| p27(5FF) | 1 200 000 | 4 200 000 | 72 | 3.50 | 39.84 |
| p28(6FF) | 2 100 000 | 6 400 000 | 96 | 3.05 | 59.71 |
| p29(7FF) | 639 996 | 2 200 000 | 72 | 3.44 | 40.42 |
| p30(8FF) | 1 920 000 | 1 700 000 | 96 | 0.89 | NA |
| p31(9FF) | 1 200 000 | 2 000 000 | 72 | 1.67 | 97.70 |
| p32(10FF) | 960 000 | 4 000 000 | 96 | 4.17 | 46.63 |
| p33(11FF) | 1 100 000 | 1 500 000 | 72 | 1.36 | 160.91 |
| p34(12FF) | 1 540 000 | 9 300 000 | 96 | 6.04 | 37.00 |
| p34(4FF) | 1 040 000 | 6 000 000 | 96 | 5.77 | 37.97 |
| p35(5FF) | 640 000 | 2 640 000 | 72 | 4.13 | 35.22 |
| p22(3FF) | 1 200 000 | 6 010 000 | 72 | 5.01 | 30.98 |
| p23(4FF) | 2 160 000 | 9 910 000 | 96 | 4.59 | 43.68 |
| p24(5FF) | 1 440 000 | 1 880 000 | 72 | 1.31 | 187.18 |
| p25(6FF) | 1 920 000 | 5 430 000 | 96 | 2.83 | 64.01 |
| p26(7FF) | 3 840 000 | 7 200 000 | 72 | 1.88 | 79.39 |
| p27(8FF) | 1 800 000 | 14 500 000 | 96 | 8.06 | 31.89 |
| p35(3FF) | 1 020 000 | 2 960 000 | 72 | 2.90 | 46.84 |
| p36(4FF) | 2 160 000 | 6 300 000 | 96 | 2.92 | 62.16 |
| p38(3FF) | 1 200 000 | 3 300 000 | 72 | 2.75 | 49.33 |
| p39(4FF) | 3 680 000 | 10 350 000 | 96 | 2.81 | 64.35 |
| p41(6FF) | 1 830 000 | 10 055 000 | 96 | 5.49 | 39.06 |
| p42(7FF) | 1 215 000 | 3 200 000 | 72 | 2.63 | 51.53 |
|  |  |  | **Average** | **3.44** | **61.88** |
|  |  |  | **STDEV** | 1.71 | 39.04 |
|  |  |  | **n (passages)** | 24 | 23 |
|  |  |  |  |  |  |
| hiPSC2 |  |  |  |  |  |
| Passage  (Feeder-free passage) | **Cells**  **initial** | **Cells**  **final** | **Time**  **(hours)** | **Multiplication within passage** | **Doubling Time. DT (hours)** |
| Passage | **Cells**  **Initial** | **Cell**  **Final** | **Time**  **(hours)** | **Multiplication within passage** | **Doubling Time (hours)** |
| p20(3FF) | 1 600 000 | 4 050 000 | 72 | 2.53 | 53.74 |
| p21(4FF) | 2 160 000 | 6 610 000 | 96 | 3.06 | 59.49 |
| p22(5FF) | 1 440 000 | 2 810 000 | 72 | 1.95 | 74.65 |
| p23(6FF) | 1 920 000 | 7 010 000 | 96 | 3.65 | 51.38 |
| p25(8FF) | 3 840 000 | 11 700 000 | 96 | 3.05 | 59.73 |
| p32(3FF) | 2 400 000 | 6 000 000 | 96 | 2.50 | 72.62 |
| p33(4FF) | 2 400 000 | 4 800 000 | 72 | 2.00 | 72.00 |
| p34(5FF) | 3 240 000 | 9 100 000 | 96 | 2.81 | 64.44 |
| p36(7FF) | 420 000 | 1 025 000 | 72 | 2.44 | 55.94 |
| p37(8FF) | 980 000 | 3 060 000 | 72 | 3.12 | 43.83 |
| p38(9FF) | 1 680 000 | 7 800 000 | 96 | 4.64 | 43.34 |
|  |  |  | **Average** | **2.89** | **59.20** |
|  |  |  | **STDEV** | 0.77 | 10.95 |
|  |  |  | **n (passages)** | 11 | 11 |
