# Supplementary material for: Xeno- and feeder-free differentiation of human pluripotent stem cells to two distinct ocular epithelial cell types using simple modifications of one method
Source: Stem Cell Res Ther. 2017 Dec 29;8:291. doi: 10.1186/s13287-017-0738-4 (PMC5747074; doi:10.1186/s13287-017-0738-4)
Supplement: Supplementary file 6 — Supplementary dataset 1. Optimization of hPSC-RPE differentiation. (DOCX 36 kb) [file 13287_2017_738_MOESM6_ESM.docx]

**Supplementary Dataset 1. Optimization of hPSC-RPE Differentiation**

Different approaches were tested to develop a functional differentiation strategy to RPE from the feeder-free hPSCs. As E8 contains large amounts of FGF and TGFβ to maintain pluripotency, it was unsuitable for RPE differentiation. Essential 6™ Medium (E6, ThermoFisher Scientific) designed for differentiation strategies and lacking the growth factors present in E8, was found insufficient for RPE differentiation as it did not promote pigmentation. Instead 15% KnockOut™ SR XenoFree CTS™ (XF-Ko-SR)-containing medium was chosen as a xeno-free media base. ***Adherent differentiation strategies*** by changing the E8 medium to the RPE differentiation medium containing 15% XF-Ko-SR and no bFGF, repeatedly led to detachment of the cell layer. Different LN-521 concentrations, combination matrix of LN-521 and col IV, plasma treatment of the cell culture plastic, weekly supplementation of culture medium with LN-521, replating to fresh matrix were tested, but adequate attachment to allow enough pigmented cell clusters for selection was not achieved***. Embryoid body (EB) differentiation in suspension*** led to loss of EBs due to disaggregation and the pigmentation rate was extremely slow**.** Addition of ROCKi or Blebbistatin increased aggregation but essentially no pigmentation was achieved. Small molecule induction with IWP-2 (Wnt inhibitor) and SB505124 (TGFβ inhibitor) during the EB differentiation did not enhance RPE differentiation rate or yield, but led to increased EB dissociation (not shown). A two-stage combination strategy of initial differentiation in suspension (day 0-5), followed by adherent culture led to desired RPE differentiation of sufficient pigmentation for selection within 35-50 days of differentiation. In addition to XF-Ko-SR medium, this differentiation strategy (**Fig. 3A**) was also tested with X-VIVO™ 10 medium (Lonza). Differentiation in X-VIVO™ 10 medium led to very high levels of pigmentation but otherwise compromised RPE morphology and epithelial properties of the final mature RPE. Average TEER value of 134 (SD 61, n=24 inserts from four separate differentiation experiments) was achieved for hESC1-RPE after 9 weeks of final culture on inserts culture. This was highly significantly lower compared to RPE differentiated in 15% XF-Ko-SR medium (p<0.01, Mann–Whitney U-test). Extreme pigmentation, ragged epithelial layers, uneven cell size, and morphology were repeatedly noted X-VIVO™ 10 medium (not shown).
