# Supplementary material for: Xeno- and feeder-free differentiation of human pluripotent stem cells to two distinct ocular epithelial cell types using simple modifications of one method
Source: Stem Cell Res Ther. 2017 Dec 29;8:291. doi: 10.1186/s13287-017-0738-4 (PMC5747074; doi:10.1186/s13287-017-0738-4)
Supplement: Supplementary file 8 — Supplementary dataset 2. Optimization of hPSC-LESC differentiation. (DOCX 42 kb) [file 13287_2017_738_MOESM8_ESM.docx]

**Supplementary Dataset 2. Optimization of hPSC-LESC Differentiation**

We first tested our previously published protocol for hPSC-LESC differentiation by blocking the TGF-β and Wnt-signaling, and activating FGF signaling [1]. 3-6-day induction with 10 µM SB-505124, 10 µM Wnt pathway inhibitor IWP-2, and 50 ng/ml bFGF, followed by plating down to col IV matrix in Cnt-30 medium, continuously led to excessive cell death or differentiation towards neuronal or other phenotypes (Additional File 9. Fig. S5). Gene expression analysis of the differentiating cells showed the feeder-free cultured cells to correspond differently to the induction compared to the hPSCs from hFF feeder system (Supplementary Fig. 5D and E). While both cell types showed decreasing expression of pluripotency marker oct-3/4, and increasing expression of eye-field transcription factor PAX6, feeder-free cultured cells showed decreasing expression pattern for BMP-4, human limbal epithelial stem cell marker ABCG2, and homeodomain transcription factor PITX2, required for corneal morphogenesis and specification from surface ectoderm. We hypothesized that in the absence of the feeder cells, mesodermal BMP-4 induction after early ectodermal induction might be beneficial in directing the differentiation toward surface ectoderm and corneal fate. Two-day induction with BMP-4 after short overnight ectodermal induction with SB-505124 and bFGF, followed by plating down to a combination matrix of col IV and LN-521 in Cnt-30 epithelial medium, led to differentiation of LESC-like epithelial cells. Col IV alone compared to the use of the combination matrix failed to support the epithelial differentiation, but instead cells detached early in the cause of differentiation. IWP-2 was omitted from the surface ectodermal induction since it caused cell death, and the removal did not have any adverse effect on the differentiation.

**References**

1. Mikhailova A, Ilmarinen T, Uusitalo H, Skottman H. Small-molecule induction promotes corneal epithelial cell differentiation from human induced pluripotent stem cells. Stem Cell Reports. 2014;2(2):219-31.
